# The Activation of Mucosal-Associated Invariant T (MAIT) Cells Is Affected by Microbial Diversity and Riboflavin Utilization *in vitro*

**DOI:** 10.3389/fmicb.2020.00755

**Published:** 2020-04-22

**Authors:** Jannike L. Krause, Stephanie S. Schäpe, Florian Schattenberg, Susann Müller, Grit Ackermann, Ulrike E. Rolle-Kampczyk, Nico Jehmlich, Arkadiusz Pierzchalski, Martin von Bergen, Gunda Herberth

**Affiliations:** ^1^Department of Environmental Immunology, Helmholtz-Centre for Environmental Research – UFZ Leipzig, Germany; ^2^Department of Molecular Systems Biology, Helmholtz-Centre for Environmental Research – UFZ, Leipzig, Germany; ^3^Department of Environmental Microbiology, Helmholtz-Centre for Environmental Research – UFZ, Leipzig, Germany; ^4^Faculty of Biosciences, Pharmacy and Psychology, Institute of Biochemistry, University of Leipzig, Leipzig, Germany; ^5^Alphaomega laboratory, Leipzig, Germany

**Keywords:** human MAIT cells, gut microbiota, folate metabolism, microbial stress, riboflavin metabolism, SIHUMIx

## Abstract

Recent research has demonstrated that MAIT cells are activated by individual bacterial or yeasts species that possess the riboflavin biosynthesis pathway. However, little is known about the MAIT cell activating potential of microbial communities and the contribution of individual community members. Here, we analyze the MAIT cell activating potential of a human intestinal model community (SIHUMIx) as well as intestinal microbiota after bioreactor cultivation. We determined the contribution of individual SIHUMIx community members to the MAIT cell activating potential and investigated whether microbial stress can influence their MAIT cell activating potential. The MAIT cell activating potential of SIHUMIx was directly related to the relative species abundances in the community. We therefore suggest an additive relationship between the species abundances and their MAIT cell activating potential. In diverse microbial communities, we found that a low MAIT cell activating potential was associated with high microbial diversity and a high level of riboflavin demand and vice versa. We suggest that microbial diversity might affect MAIT cell activation via riboflavin utilization within the community. Microbial acid stress significantly reduced the MAIT cell activating potential of SIHUMIx by impairing riboflavin availability through increasing the riboflavin demand. We show that MAIT cells can perceive microbial stress due to changes in riboflavin utilization and that riboflavin availability might also play a central role for the MAIT cell activating potential of diverse microbiota.

## Introduction

The intestinal microbiota, which clearly outnumbers the microbiota in other human habitats, shapes the immune system in various ways (Thaiss et al., 2016; Levy et al., 2017). In addition to its immunomodulatory properties, the intestinal microbiota is essential in various processes such as food digestion, colonization resistance and the synthesis of short chain fatty acids (SCFA) and vitamins (Brestoff and Artis, 2013; Sender et al., 2016; Belkaid and Harrison, 2017; Levy et al., 2017). Environmental factors, such as diet, chemicals or drugs, influence the intestinal microbiota (Goodrich et al., 2016) and can thus increase the risk of disease initiation (Forbes et al., 2016). A reduction in microbial diversity, together with an increasing presence of mucosal-associated invariant T (MAIT) cells in the inflamed intestinal or adipose tissue have been reported from patients with inflammatory bowel diseases (IBD) ulcerative colitis and Crohn’s disease (Serriari et al., 2014) or obesity (Magalhaes et al., 2015; Chiba et al., 2018).

Especially in IBD, the microbial diversity is unambiguously reduced. Also the number of *Firmicutes* and *Bacteroides* is decreased, while the frequency of *Actinobacteria* and *Proteobacteria* is increased. These changes in microbial diversity and composition as well as the acid fecal pH due to the faster gut transit time change the metabolic profile of intestinal microbiota (Moco et al., 2014) and might affect MAIT cells that accumulated in the intestinal mucosa of IBD patients (Chiba et al., 2018).

The majority of MAIT cells express the semi-invariant alpha chain 7.2 in their T-cell receptor (TCR), which is encoded by the TRAV1-2 gene. These TRAV1-2^+^ MAIT cells are considered an innate-like T cell subset with effector memory-like phenotype (Dusseaux et al., 2011; Gherardin et al., 2016). The majority of these cells recognize microbial metabolites from the riboflavin biosynthesis pathway, but a small fraction of these TRAV1-2^+^ MAIT cells also recognizes folate derivates after presentation on major histocompatibility complex I (MHC-I) related protein 1 (MR1) *in vitro* (Kjer-Nielsen et al., 2012; Corbett et al., 2014; Eckle et al., 2015; Gherardin et al., 2016). It has been shown that especially the riboflavin precursors 5-(2-oxopropylideneamino)-6-D-ribitylaminouracil (5-OP-RU) and 5-(2-oxoethylideneamino)-6-D-ribitylaminouracil (5-OE-RU) activate MAIT cells, whereas the folate derivates 6-formylpterin (6-FP) and N-acetyl-6-formylpterin (Ac-6-FP) inhibit MAIT cell activation *in vitro* (Kjer-Nielsen et al., 2012; Corbett et al., 2014). Moreover, MAIT cells can be activated independent of MR1 via cytokines (Ussher et al., 2014; van Wilgenburg et al., 2016). Microbial infections, but not commensal microbiota, are considered to trigger inflammation and thus induce the entire repertoire of MAIT cell effector function, but *in vivo* evidence is pending (Tastan et al., 2018). Nevertheless, MAIT cells are not able to distinguish commensal bacteria from pathogenic bacteria due to antigen recognition, and very little is known about the interaction of MAIT cells and the commensal microbiota (Berkson and Prlic, 2017). After activation, MAIT cells immediately produce effector molecules such as tumor necrosis factor (TNF), interferon gamma (IFNγ) and cytotoxic molecules like perforins or granzymes (Martin et al., 2009; Kurioka et al., 2015). In the human body, MAIT cells reside at barrier sites e.g., in the gut lamina propria (Treiner et al., 2003), the lung (Hinks, 2016), the female genital tract (Gibbs et al., 2017) and the skin (Teunissen et al., 2014). In addition, they are very common in the liver (Dusseaux et al., 2011) and account for to up to 10% of circulating T cells in peripheral blood (Tilloy et al., 1999). The localization of MAIT cell in combination with their ability to recognize and respond to microbial metabolites suggests a key role in host microbiota immune homeostasis and underlines their contribution to fight against infectious diseases.

Recent research has focused on the MAIT cell activating potential of individual commensal and pathogenic microorganisms from the human gut (Le Bourhis et al., 2013; Dias et al., 2017; Tastan et al., 2018). However, in the human body, MAIT cells encounter diverse microbiota and the response of MAIT cells to microbial communities rather reflects the physiologic situation. Thus, in this study we investigate the response of MAIT cells to microbial communities. Therefore, we first used the extended simplified human microbiota (SIHUMIx) model community to analyze the contribution of individual community members on MAIT cell activation. Second, we determined if microbial stress, here a short-term acid stress, affects the community composition or metabolism of SIHUMIx and thereby MAIT cell activation. Third, we investigated the MAIT cell response to microbiota with high diversity like fecal and colonic microbiota.

## Materials and Methods

### The Model Community SIHUMIx

The extended simplified intestinal human microbiota (SIHUMIx) community was used to investigate the interaction of intestinal bacterial communities and MAIT cells. This model community shows major features of a human intestinal community (Becker et al., 2011) and allows the reproducible cultivation (Krause et al., 2020). SIHUMIx comprises of eight bacterial species *Anaerostipes caccae* (DSM 14662), *Bacteroides thetaiotaomicron* (DSM 2079), *Bifidobacterium longum* (NCC 2705), *Blautia producta* (DSM 2950), *Clostridium butyricum* (DSM 10702), *Clostridium ramosum* (DSM 1402), *Escherichia coli* K-12 (MG1655), and *Lactobacillus plantarum* (DSM 20174).

### Cultivation of Bacteria

#### Cultivation of SIHUMIx Single Strains

All bacteria strains were anaerobically cultivated using the Hungate technique in Brain-Heart-Infusion (BHI) medium (Supplementary Table S1). After inoculation, the bacteria were incubated at 37°C and 175 rpm shaking for 24 h. Then, bacteria were fixed for MAIT cell stimulation assays or kept at room temperature for a maximum of 7 d for strain maintenance. For the purpose of bioreactor inoculation, the single strain bacteria were cultivated for 72 h in BHI medium.

#### Continuous Cultivation of SIHUMIx and Human Fecal Communities

To investigate the stimulation capacity of SIHUMIx under normal and stress conditions the SIHUMIx community was continuously cultivated in a Multifors 2 bioreactor (Infors, Switzerland, *N* = 2) as described in Krause et al. (2020) using complex intestinal medium [CIM, Supplementary Table S2 (Krause et al., 2020)]. In addition, we cultivated human fecal microbiota alike the SIHUMIx community in triplicate bioreactors. In brief, growth conditions should reflect the colon of a healthy individual. After the sterile run, the bioreactors were inoculated with an equal cell number of SIHUMIx strains or 1 mL fecal enrichment culture. Bacteria were cultivated under unimpeded culture conditions until day 14 (SIHUMIx) or day 16 (fecal microbiota). SIHUMIx additionally was exposed to an acid stress, therefore in duplicate bioreactors the pH was reduced from 6.5 to 5.5 on day 4 for 24 h. After sampling on day 5, the pH was reset to the original pH of 6.5 until the end of the cultivation.

#### Continuous Cultivation of Swine Colonic Bacteria

Colonic bacteria from two different, co-housed swine were continuously cultivated in duplicate multifors 2 bioreactors (Infors, Bottmingen Switzerland, *N* = 4) until metabolic stability was assumed (10× bioreactor turnover: day 21). Bacteria were cultivated in complex intestinal medium adapted to swine [swine CIM, Supplementary Table S3, adaption from (Tanner et al., 2014; McDonald et al., 2015)]. The cultivation temperature was set to 37°C and pH was adjusted to 6.5. Prior to inoculation, a sterile run was conducted under experimental conditions. We inoculated with the supernatant of 0.5 g colon content/vessel suspended in pre-warmed CIM. Colon content from pig 1 was used for bioreactor A and B; colon content from pig 2 was used to inoculate bioreactor C and D. After 24 h continuous cultivation was started continuous cultivation at a dilution rate of *D* = 0.02 [equal to a retention time of 48 h; (Wilfart et al., 2007)].

### Bacteria Fixation

Bacterial cells were harvested (3.200 g, 5 min, 4°C) and fixed with 1% of formaldehyde for 1 min to preserve the cell structure and prevent bacteria lysis during freezing and thawing. Afterward, cells were washed three times with phosphate buffered saline (PBS, 140 mM NaCl, 10 mM Na_2_HPO_4_, 7 mM KH_2_PO_4_) to dilute out the formaldehyde. The cell number was determined using a Beckman Coulter Multisizer 3 cell counting system (Beckman Coulter, Brea, United States) and the cell number was adjusted to 3 × 10^9^ cells/mL in IMDM medium (IMDM supplemented with 10% fetal calf serum, 25 mM HEPES, 50 μM β-mercaptoethanol and 100 U/mL Pencillin/Streptomycin). Bacteria pellets were frozen with IMDM supernatant at −80°C.

### Purification of Peripheral Blood Mononuclear Cells

Blood from healthy donors was obtained from the blood donation service at the university hospital Leipzig, Germany. PBMCs were purified by gradient centrifugation on Ficoll-paque plus (GE Healthcare, Chicago, United States). PBMCs were gradually frozen in FCS with 10% DMSO at −80°C and stored at −150°C until use.

### MAIT Cell Stimulation

The day before stimulation PBMCs were thawed and 1 × 10^6^ of live PBMCs per well were seeded into 96-well plates. PBMCs were incubated over-night at 37°C and 5% CO_2_. For stimulation, frozen bacteria pellets were mixed vigorously, diluted in IMDM, and used directly in a total volume of 200 μL.

1 × 10^6^ PBMCs were stimulated with 25 bacteria per cell (BpC) of all SIHUMIx single strains. The SIHUMIx 1:1 mix was generated by mixing equal cell numbers of the SIHUMIx strains. We used 25 BpC from the SIHUMIx 1:1 mix for stimulation. To calculate the average percentage of MAIT cell activation, we summarized the percentage of activated MAIT cells after single strain stimulation and divided by the number of strains.

PBMCs were stimulated with 200 BpC of complex communities that were cultivatied in the bioreactor, like SIHUMIx, the colonic and the fecal communities. The negative control remained unstimulated; the positive control was stimulated with 20 BpC *E. coli* K12.

To compare sample diversity, 200 BpC of *E. coli*, the SIHUMIx, the fecal and the colonic community were used for stimulation. To achieve an average MAIT cell response to SIHUMIx and the colonic communities, we pooled the bioreactor samples of both bioreactors on day 13 and day 14, respectively (recovered SIHUMIx communities). Likewise, we pooled all colonic communities A, B, C, and D on day 21 for MAIT cell stimulation assays.

MR1 antibody was incubated one hour before with PBMCs. Then bacteria were added for MAIT cell stimulation.

After two hours of stimulation 10 μg/mL Brefeldin A was added to prevent cytokine release. After a total of 6 h PBMCs were harvested for surface (CD3, CD8a, CD161, Va7.2) and intracellular staining (CD69, TNF, IFNg) followed by FACS analysis (FACS Canto II, Becton Dickinson and Company, Franklin Lakes, United States). Discrimination of dead cells was done by staining with Fixable Viability Dye eFluor 506. Antibodies were obtained from Biolegend and eBioscience (Supplementary Table S4). Data analysis was done with FlowJo v10 software. Data evaluation and hypothesis testing was done with GraphPad PRISM v7.04 software using one-way ANOVA.

### Quantitation of Riboflavin in Bacterial Culture Supernatants

Supernatant samples were thawed at 37°C for 10 min. We extracted the metabolites with five volumes of a methanol:acetonitrile:water (2:3:1) mixture and added 10 μL internal standard. After addition of five volumes of extraction solvent samples were vortexed for 5 min and afterward sonicated in an ultrasound bath for 5 min. Debris was pelleted by centrifugation (14.000 rpm, 5 min, RT) and the supernatant was transferred to a fresh tube. The extract was dried in a SpeedVac vacuum concentrator (Eppendorf, Hamburg, Germany) and resuspended in 100 μL of mix of running solvent A and running solvent B (1:1).

For LC-MS/MS measurement 10 μL of the resuspended extract were injected into a HPLC-MS-System (RSLC Ultimate 3000 Thermo Fisher coupled with Q-Trap 5500 AB Sciex). Metabolites were separated on ACQUITY UPLC BEH 300 C18 (1,7 μm, Waters, Milford, United States) with a flow rate of 0.3 mL/min in a gradient of running solvent A (0.1% formic acid in water) and running solvent B (0.1% formic acid in methanol): 0.5 min 100% A, 0.6–4 min 0–100% B, hold 3 min, 3 min 100% A. The Q-Trap was set up to positive MRM mode (Riboflavin MRM: parent ion: 377, product ions: 243, 198, and 172; Internal Standard MRM: parent ion: 383, product ions: 249, 202, and 175). Bar plots were generated with the GraphPad PRISM v7.04 software.

### Quantitation of Folate in Bacterial Culture Supernatants

Supernatant of bacterial cultures was used for the measurement of total folate concentration with the electrochemiluminescence immunoassay Elecsys Folate (Roche, Basel, Switzerland) according to manufacturer’s instructions.

### Metaproteome and Proteome Analysis

Bacteria pellets were thawed and dissolved in the 1000 μL lysis buffer (10 mM Tris–HCl, NaCl 2 mg/mL, 1 mM PMSF, 4 mg/mL SDS). Cells were disrupted by 1. Bead beating (FastPrep-24, MP Biomedicals, Santa Ana, United States: 5.5 ms, 1 min, 3 cycles), 2. 15 min incubation at 60°C (Thermomixer comfort 5355, Eppendorf, Germany) and 3. Ultra-sonication UP50H, Hielscher, Teltow, Germany; cycle 0.5, amplitude 60%). Protein concentration was determined with bicinchoninic acid assay according to the user manual (Pierce BCA Protein Assay Kit, Thermo Fisher Scientific, Waltham, United States). 100 μg of protein were precipitated overnight in acetone 1:5 (V/V) at -20°C and centrifuged (10 min, 14,000 × *g*). The precipitate was used for SDS-PAGE analysis, in-gel digestion and protein purification with ZipTip treatment (Starke et al., 2016).

Measurement was performed as described (Krause et al., 2020). 5 μg peptide lysate was injected into nanoHPLC (UltiMate 3000 RSLCnano, Dionex, Thermo Fisher Scientific, Waltham, United States). Peptide separation was performed on a C18-reverse phase trapping column (C18 PepMap100, 300 μm × 5 mm, particle size 5 μm, nano viper, Thermo Fischer Scientific, Waltham, United States), followed by a C18-reverse phase analytical column (Acclaim PepMap^§^ 100, 75 μm × 25 cm, particle size 3 μm, nanoViper, Thermo Fisher Scientific, Waltham, United States). Mass spectrometric analysis of peptides was performed on a Q Exactive HF mass spectrometer (Thermo Fisher Scientific, Waltham, United States) coupled to a TriVersa NanoMate (Advion, Ltd., Harlow, United Kingdom) source in LC chip coupling mode. LC gradient, ionization mode, and mass spectrometry mode are described (Haange et al., 2019).

Raw data were processed with Proteome Discoverer (v 2.2, Thermo Fischer Scientific, Waltham, United States). Search settings for Sequest HT search engine were set to: Trypsin (Full), Max. Missed Cleavage: 2, precursor mass tolerance: 10 ppm, fragment mass tolerance: 0.02 Da. Protein grouping was enabled, with protein group requiring at least one unique peptide. For single species the protein coding sequences of the eight SIHUMIx strains were downloaded from UniProt^1^. For SIHUMIx protein coding sequences of all eight were combined and used as database resulting in 29,558 protein sequences. For complex microbiota protein coding sequences of all “bacteria” were downloaded from UniProt (13.05.2017; http://www.uniprot.org/) resulting in 15,214,675 protein coding sequences. Protein identification was performed as descried (Schäpe et al., 2019). In brief, the false discovery rates (FDR) were determined with the node Percolator (Käll et al., 2007) embedded in Proteome Discoverer (v 2.2) and we set the FDR threshold at peptide and protein level at 5%. Only protein groups were assigned that explains at least one unique identified peptide.

*GhoastKOALA* was used to assign KO numbers of KEGG to identified functions of identified protein sequences. Visualization and statistical analysis were done with GraphPad Prism (v. 8.0.2) using unpaired multiple *t*-tests per row.

### 16 S rRNA Gene Analysis

For DNA extraction, bacteria pellets were thawed and one volume of bacteria slurry was mixed with 30 volumes of sterile 10% Chelex (wt/vol) solution. Samples were incubated in a ThermoMixer (Eppendorf, Hamburg, Germany) at 95°C for 45 min and 1000 rpm shaking. Afterward the suspension was centrifuged for 3 min at 11,000 *g* and the supernatant, containing the DNA, was transferred into a fresh, sterile tube and stored at -20°C. High throughput 16S amplicon generation, sequencing and analysis was done by StarSeq GmbH (Mainz, Germany). The 16S gene region V3 to V4 was amplified with the primers 341F and 806bR, sequencing was performed on an Illumina MiSeq DNA sequencer (Illumina, San Diego, United States).

### Microbial Flow Cytometry

The bacteria suspension was centrifuged (3,200 × g, 10 min, and 4°C) and cells were fixed in 2% formaldehyde [stock: 8% formaldehyde pH 7, diluted with PBS (6 mM Na_2_HPO_4_, 1.8 mM NaH_2_PO_4_ and 145 mM NaCl in bi-distilled water, pH 7)] at RT for 30 min. The bacteria were pelleted (3,200 × g, 10 min, and 4°C) and resuspended in 70% ethanol for long-term storage at −20°C.

After a minimum of one day at −20°C, single strain bacteria and SIHUMIx were stained with 0.24 μM 4′,6-di-amidino-2-phenyl-indole (DAPI, Sigma-Aldrich, St-Louis, United States) overnight according to the protocol from Koch et al. (2013). The fecal and colonic bacteria were homogenized by ultra-sonication and stained with 0.68 μM 4′,6-di-amidino-2-phenyl-indole (DAPI, Sigma-Aldrich, St-Louis, United States) overnight. Measurement and data analysis were performed as in Krause et al. (2020).

### Statistical Analysis

Bar plots report the mean and standard deviation. Comparison of groups were done using GraphPad Prism version 8.3.0 (La Jolla, CA, United States) using Student’s t-test or ordinary one-way ANOVA for unpaired data with Dunnett’s or Tukey *post hoc* test for multiple comparisons as specified in the figure legends. Significance was defined at *P* < 0.05. The calculation of Bray-Curtis-Dissimilarities was done in R using the metaMDS function from the vegan package (Dixon, 2003), data were visualized in a heatmap using the heatmap.2 function.

## Results

### The Model Community SIHUMIx Comprises of MAIT Cell Activating and Non-activating Strains

**FIGURE 1:**
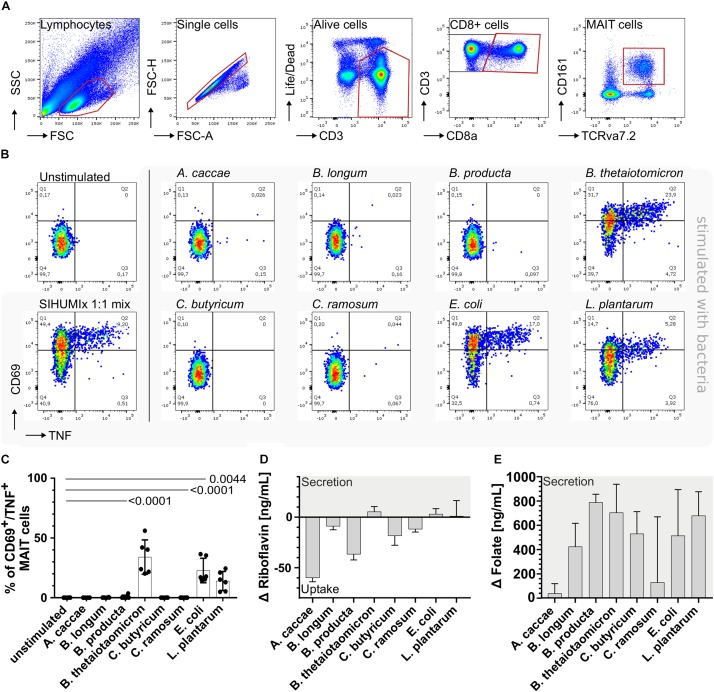
Stimulation of mucosal-associated T (MAIT) cells with individual community members of SIHUMIx. **(A)** We identified MAIT cells as alive CD3^+^, CD8^+^, CD161^+^, and TCRvα7.2^+^ from single cell lymphocytes. **(B)** We defined activated MAIT cells by the expression of CD69^+^/TNF^+^ and show exemplary dot plots of unstimulated MAIT cells and MAIT cells after stimulation with the individual SIHUMIx strains [25 bacteria per cell (BpC)]. Moreover, we mixed the individual SIHUMIx strains in equal cell number (SIHUMIx1:1 mix) for stimulation (25 BpC). **(C)** Bar plots quantify the percentage of activated CD69^+^/TNF^+^ MAIT cells after stimulation with the individual SIHUMIx strains compared to the unstimulated control. Bars represent mean ± sd, *n* = 6, ordinary one-way ANOVA, unpaired, Dunnett’s multiple comparison test. The riboflavin **(D)** and folate **(E)** concentrations were measured in the culture supernatant of SIHUMIx after cultivation in Brain-Heart Infusion (BHI) medium [ng/mL]. Bars represent mean ± sd, *n* = 3. Positive changes indicate vitamin secretion, negative values indicate vitamin uptake.

To characterize the response of MAIT cells to intestinal bacterial communities and the contribution of individual strains, we utilized the extended simplified human microbiota model community (SIHUMIx). SIHUMIx is a defined bacterial community of eight species commonly found in the human intestine: *A. caccae*, *B. thetaiotaomicron*, *B. longum*, *B. producta*, *C. butyricum*, *C. ramosum*, *E. coli* K-12 and *L. plantarum* (Becker et al., 2011). First, we evaluated the activating potential of the individual community members and therefore individually stimulated peripheral blood mononuclear cells (PBMCs) with these bacterial strains (Figures 1A–D, *n* = 6). In this study, all bacteria were treated with formaldehyde and stored at −80°C prior to PBMC stimulation. Nevertheless, the cellular structure of the bacteria was largely preserved and bacterial lysis during the freeze-thaw-cycle was prevented (total and viable bacteria cell numbers: Supplementary Figure S1 and viability staining images: Supplementary Figure S2).

Furthermore, we identified TRAV1-2^+^ MAIT cells by the expression of CD3^+^, CD8a^+^, CD161^+^, and the TCR Vα 7.2^+^ surface receptors within the alive single cell lymphocytes (hereafter called MAIT cells, Figure 1A). CD69^+^/TNF^+^ expressing MAIT cells were defined as activated MAIT cells (Figure 1B). Upon co-incubation, the bacterial strains *B. thetaiotaomicron, E. coli*, and *L. plantarum* activated MAIT cells in decreasing order and significantly increased the percentage of CD69^+^/TNF^+^ MAIT cells compared to the unstimulated control (Figure 1C, one-way-ANOVA, *n* = 6). *B. thetaiotaomicron* e.g., activated 34.0% of MAIT cells (*P* < 0.0001). The remaining strains *A. caccae*, *B. longum*, *B. producta*, *C. butyricum*, and *C. ramosum* were not able to activate MAIT cells.

Resent research showed that MAIT cell activating bacteria possess at least the enzyme ribD/G from the riboflavin biosynthesis pathway (Corbett et al., 2014; Kurioka et al., 2015; Soudais et al., 2015). Other *in vitro* studies proved that the majority of MAIT cells recognize riboflavin metabolites and after recognition become activated (Corbett et al., 2014). Nevertheless, a small proportion of MAIT cells recognizes folate derivates (Gherardin et al., 2016). Since these metabolites are difficult to quantify, we measured the riboflavin and folate concentration in the culture supernatant as a proxy. After blank subtraction, a positive Δ vitamin concentration indicated vitamin secretion into the culture medium, whereas a negative value indicated vitamin uptake from the culture medium.

Exclusively for the MAIT cell activating bacterial strains, *B. thetaiotaomicron, E. coli* and *L. plantarum*, we observed positive Δ riboflavin concentrations (Figure 1D, raw data: Supplementary Table S5 and Supplementary Figure S3A) suggesting the ability of riboflavin biosynthesis and secretion. *B. thetaiotaomicron* had the highest Δ riboflavin concentration combined with the highest potential to activate MAIT cells. Both, the ability to activate MAIT cells and the Δ riboflavin concentration in the culture supernatant were lower for *E. coli* and the lowest for *L. plantarum* compared to *B. thetaiotaomicron.* Additionally, we observed negative Δ riboflavin concentrations for all non-activating bacterial strains indicating riboflavin uptake from the culture medium (Figure 1D). The Δ folate concentrations were positive after blank subtraction for all bacterial strains independent of their MAIT cell activating potential and exceeded the riboflavin concentration (Figure 1E, raw data: Supplementary Table S6 and Supplementary Figure S3B). Our results indicate that the MAIT cell activating SIHUMIx strains, *B. thetaiotaomicron, E. coli*, and *L. plantarum*, are capable of riboflavin biosynthesis and secretion, whereby the non-activating SIHUMIx strains seem not to be able to synthesize riboflavin and thus might take up riboflavin from the medium.

**FIGURE 2:**
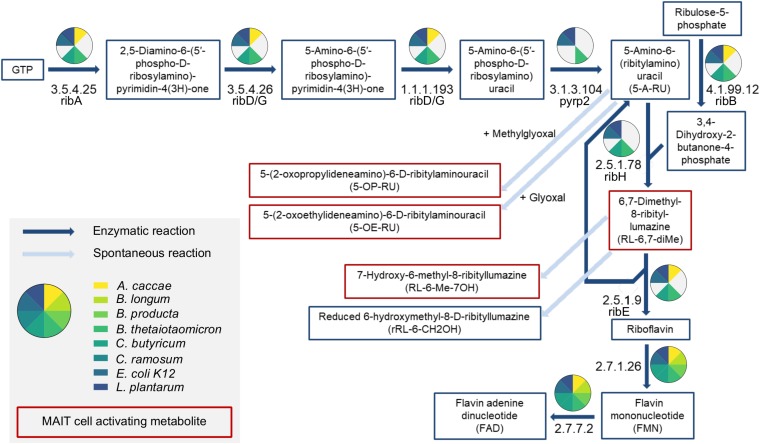
Riboflavin biosynthesis pathway analysis of SIHUMIx bacterial strains. Enzymes from the riboflavin biosynthesis pathway are coded by their EC number. The colors used in the pie charts indicate detected proteins of corresponding bacterial strains. Metabolites that activate MAIT cells after presentation on MHC I related protein 1 (MR1) are marked in red.

We next analyzed the enzymatic repertoire to synthesize riboflavin of the SIHUMIx strains to confirm our findings regarding the ability to synthesize riboflavin and/or folate. Therefore, we combined shotgun proteomics with a proteome and genome based data base search in the UniProt and KEGG database (Figure 2). We detected protein abundance levels assigned to riboflavin biosynthesis with proteomics in *B. thetaiotaomicron*, *E. coli*, and *L. plantarum*. Using the database search, we also found proteins from the riboflavin biosynthesis pathway for *A. caccae* and *C. butyricum* (Supplementary Table S7). The remaining bacterial strains did not possess enzymes from the riboflavin biosynthesis pathway. However, only the MAIT cell activating bacterial strains *B. thetaiotaomicron*, *E. coli*, and *L. plantarum* proved to have all enzymes for riboflavin biosynthesis. Moreover, we detected proteins for riboflavin uptake (except *C. ramosum*) and riboflavin conversion into the cofactors flavin mononucleotide (FMN) and flavin adenine dinucleotide (FAD) in all SIHUMIx strains expect (Figure 2). Indeed, the MAIT cell activating SIHUMIx strains as only had the necessary enzymes for riboflavin biosynthesis. In contrast, the non-activating SIHUMIx strains possessed incomplete riboflavin biosynthesis pathways combined with the enzymatic equipment for riboflavin uptake and conversion. With regard to folate, we observed an increase in Δ folate combined with a close to full enzyme coverage of the folate biosynthesis pathways for all SIHUMIx strains, except *C. ramosum*, indicating the ability to produce and secret folate (Supplementary Table S8 and Supplementary Figure S4).

**FIGURE 3:**
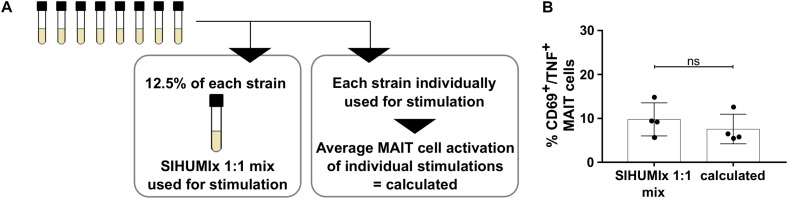
Contribution of individual SIHUMIx strains to the MAIT cell activating potential. **(A)** Study design: Left – Mixture of individual SIHUMIx strains in equal cell numbers (SIHUMIx 1:1 mix) were used for stimulation. Right – Individual SIHUMIx strains were used for stimualtion. **(B)** We compared the MAIT cell activating potential of the SIHUMIx 1:1 mix community to the calculated MAIT cell activating potential based on the individual activating potential of each SIHUMIx strain (mean ± sd, *n* = 4, unpaired *t*-test).

To understand the contribution of individual bacterial strains to the MAIT cell activating potential of bacterial communities, we stimulated MAIT cells with an artificial SIHUMIx community, the SHIUMIx 1:1 mix. In this SIHUMIx 1:1 mix, all strains were mixed from single strain cultures at equivalent abundances (SIHUMIx 1:1 mix, Figure 3A). We compared the MAIT cell activating potential of SIHUMIx 1:1 mix to a calculated value. This calculated value represents the average MAIT cell response after MAIT cell stimulation with the individual SIHUMIx strains (Figure 1A). The MAIT cell activating potential of SIHUMIx 1:1 mix was equal to the calculated MAIT cell response (Figure 3B, *n* = 4, unpaired t-test, ns). Therefore, we assume a correlation between the relative species abundances in the community and the MAIT cell activating potential of the community.

### Acid Stress Affects the MAIT Cell Activating Potential by Altering Microbial Riboflavin Metabolism

MAIT cells and microbiota convene at the body’s barrier sites, where environmental factors or stressors can affect the microbiota (Goodrich et al., 2016). We aimed to investigate if microbial stress can affect the MAIT cell activating potential of microbiota and thereby can directly influence MAIT cell activation. Since patients with active ulcerative colitis can show a lower colonic pH as part of the disease (Nugent et al., 2001; Moco et al., 2014), we cultivated the SIHUMIx model community *in vitro* and induced an acute acid stress of pH 5.5 (Krause et al., 2020), experimental set-up: Supplementary Figure S5). Thereafter, we compared the MAIT cell activating potential of the unstressed and the acid stressed SIHUMIx community.

**FIGURE 4:**
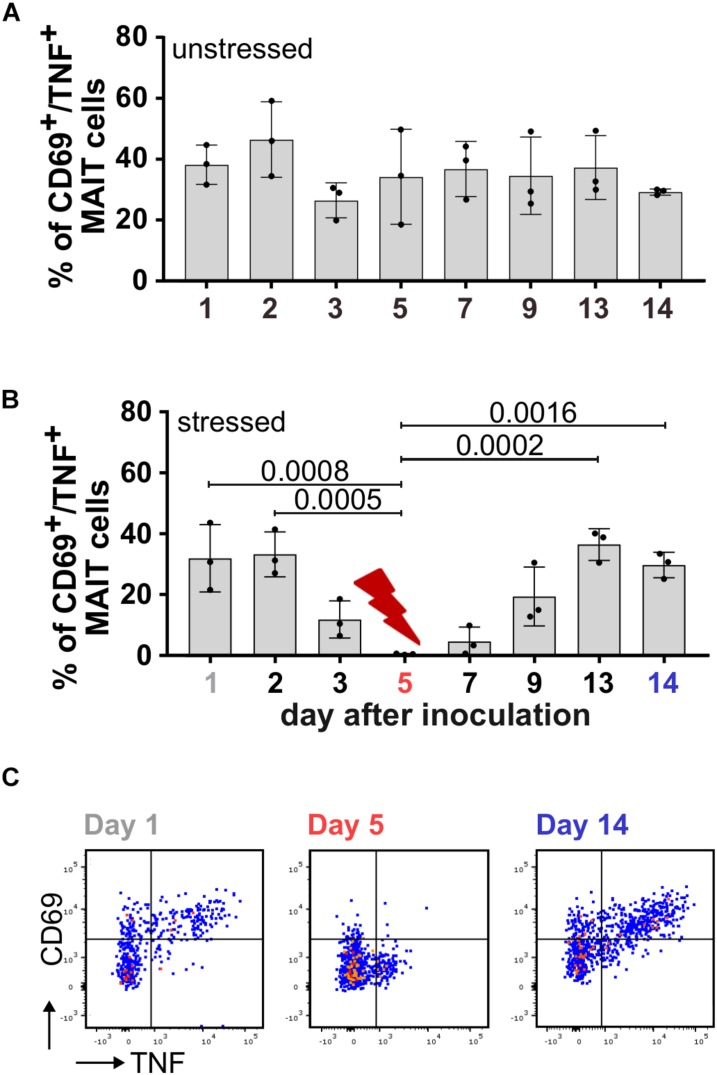
The MAIT cell activating potential of SIHUMIx is lost during acid stress. We cultivated SIHUMIx community in bioreactors (*n* = 4) for 14 d. On day 5, bacteria were exposed to a short-term acid stress (*n* = 2), whereas others remained untreated (*n* = 2). **(A)** Bar plots quantify the percentage of CD69^+^/TNF^+^ activated MAIT cells after stimulation with pooled SIHUMIx communities from unstressed bioreactors and **(B)** bioreactors that were exposed to a short-term acid stress on days 1, 2, 3, 5, 7, 9, 13, and 14. Bars represent mean ± sd (*n* = 3, unpaired data, ordinary one-way ANOVA, Tukey *post hoc* test for multiple comparisons). **(C)** We show exemplary dot plots of CD69^+^/TNF^+^ activated MAIT cells after stimulation with SIHUMIx from the stressed bioreactors on day 1 (initial), day 5 (acid stress) and day 14 (recovered) with 200 bacteria per cell (BpC).

During adaptation to the culture conditions in the bioreactor system on day 1 and day 2, the MAIT cell activating potential of SIHUMIx was highest and dropped slightly on day 3 in both, the unstressed and the stressed SIHUMIx community (Figure 4). In the unstressed SIHUMIx, the MAIT cell activating potential remained constant until the end of the experiment after day 3 (Figure 4A, *n* = 3, one-way ANOVA). Under acid stress SIHUMIx lost the potential to activate MAIT cells on day 5 (Figure 4B,C, *n* = 3, one-way ANOVA, *P* are listed in Supplementary Table S9). After resetting the pH to the original value, the MAIT cell activating potential recovered to the initial MAIT cell activating potential on day 13 and day 14 (Figure 4B,C, *n* = 3).

**FIGURE 5:**
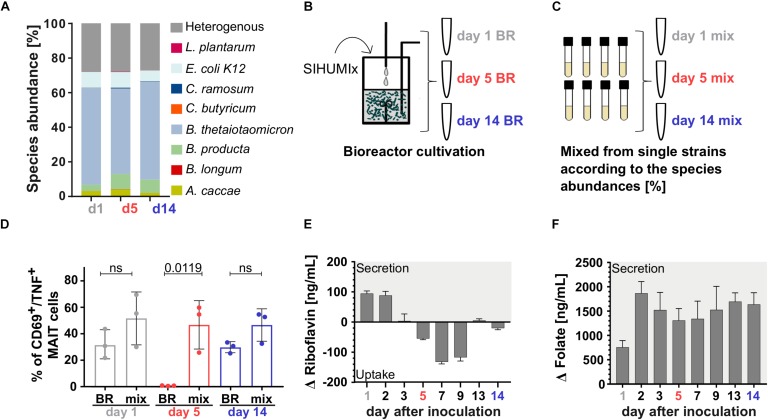
Acid stress mainly affected microbial riboflavin utilization and thereby MAIT cell activation. **(A)** The relative species abundances of SIHUMIx on day 1, day 5, and day 14 observed by metaproteomics was similar. **(B)** We stimulated MAIT cell with SIHUMIx communities on days 1, 5, and 14 cultivated in the bioreactor (BR) and **(C)** with mix communities based on the relative species abundances observed by metaproteomics. These were prepared from individual cultures on days 1, 5, and 14. **(D)** The unstressed bioreactor communities (BR) on day 1 and day 14 had a similar MAIT cell activating potential as their corresponding mix communities (mix), whereas on day 5 the BR community under acid stress significantly lost its MAIT cell activating potential (mean ± sd, *n* = 3, unpaired *t*-test). Quantitation of **(E)** riboflavin and **(F)** folate in the culture supernatant of SIHUMIx after bioreactor cultivation [ng/mL]. Bars represent mean ± sd, *n* = 3. Positive changes indicate vitamin secretion, negative values indicate vitamin uptake.

Changes in the relative species abundances of SIHUMIx might cause the reduced MAIT cell activating potential under acid stress. Therefore, we performed metaproteomics to elucidate the community composition. Based on the relative species abundances day 1, day 5, and day 14 segregated in a principal component analysis (Supplementary Figure S6) although the community compositions on day 1, day 5 and day 14 were similar (Figure 5A and Supplementary Table S10). Nevertheless, our data revealed an increase in the low abundant bacterial strains *B. longum*, *C. ramosum* and *L. plantarum* under acid stress (day 5) compared to the communities on day 1 and day 14 (Supplementary Figure S7).

From our findings, we expected an additive relation between the relative species abundances (community composition) and the MAIT cell activating potential in the model community SIHUMIx (Figure 3B). Thus, we compared the activating potential of bioreactor grown SIHUMIx communities on day 1, day 5, and day 14 (Figure 5B, day 1/5/14 BR) to SIHUMIx mix communities (Figure 5C, day 1/5/14 mix). We aimed to test if the increased relative species abundances of *B. longum*, *C. ramosum*, and *L. plantarum* were causative for the reduced MAIT cell activating potential under acid stress. The mix communities were generated from single strain cultures equal to the community composition on day 1, day 5, and day 14 observed by metaproteomics (Figure 5A and Supplementary Table S10).

The acid stressed SIHUMIx community (day 5) from the bioreactor had a significantly lower potential to activate MAIT cells compared to the analogous mix-community (Figure 5D, unpaired *t*-test, *P* = 0.0199). In contrast, the unstressed bioreactor communities on day 1 and day 14 as well as their corresponding mix-communities, showed a similar MAIT cell activating potential (Figure 3D, unpaired *t*-test, ns). These results suggest that acute acid stress barely affected the community composition and that the slight changes in community composition did not account for the reduced MAIT cell activating potential under acid stress.

Since riboflavin and folate metabolites might determine the MAIT cell activating potential, we measured the concentration of both these vitamins as proxy for the metabolites. After blank subtraction, we observed high initial Δ riboflavin concentrations during adaptation, which significantly dropped on day 3 and equaled zero. Under acute acid stress, the Δ riboflavin concentration significantly dropped further and reached a minimum on day 7. After the stress ended, the Δ riboflavin concentration recovered to zero and became similar to the concentration on day 3 (Figure 5E and Supplementary Table S11, one-way ANOVA: *P* are listed in Supplementary Table S12). In contrast to riboflavin, folate was detected in high concentrations independent of the acid stress (Figure 5F and Supplementary Table S11, one-way ANOVA: *P* are listed in Supplementary Table S13). Furthermore, we considered global functional effects, but we did not observe other changes in the communities’ metabolism related to the acid stress (Supplementary Figure S8). We found, that microbial acid stress barely affected the community composition and metabolism of SIHUMx but altered the riboflavin utilization toward an increased riboflavin uptake from the medium.

### The MAIT Cell Activating Potential of Diverse Colonic Communities

**FIGURE 6:**
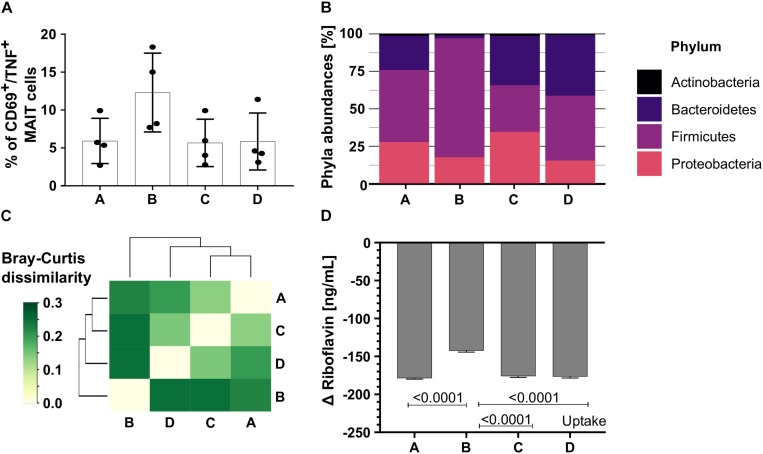
The MAIT cell activating potential of diverse colonic microbiota. We cultivated four colonic communities in continuous bioreactors. **(A)** Bar plots quantify the percentage of CD69^+^/TNF^+^ activated MAIT cells after stimulation with the colonic communities A, B, C, and D (200 bacteria per cell). Bars represent mean ± sd, *n* = 4, ordinary one-way ANOVA, unpaired, Tukey *post hoc* test for multiple comparisons. **(B)** Phyla abundances of the colonic communities A, B, C, and D based on metaproteomics. **(C)** The matrix illustrates the Bray-Curtis dissimilarities between the communities A, B, C, and D. Dark green indicates high dissimilarity between community pairs, light green indicates low dissimilarity. **(D)** The riboflavin concentration was measured in the culture supernatant of all four colonic communities [ng/mL]. Bars represent mean ± sd, *n* = 3. Positive changes indicate riboflavin secretion, negative values indicate riboflavin uptake.

To test the impact of more diverse communities on MAIT cell activation, we continuously cultivated colonic communities from swine colon content in bioreactors A, B, C, and D (community A, B, C, and D). The colonic communities A, C, and D had a similar MAIT cell activating potential (Figure 6A, *n* = 4, *A* = 5.9%, *C* = 5.67%, *D* = 5.9% CD69^+^/TNF^+^ MAIT cells, one-way ANOVA, ns). In contrast, the potential to activate MAIT cells was almost twice as high for community B (Figure 6A, *n* = 4, 12.3% CD69^+^/TNF^+^ MAIT cells, one-way ANOVA, ns).

Since the community composition in the model community SIHUMIx on the one hand and microbial riboflavin metabolism on the other hand correlated with the MAIT cell activating potential, we performed metaproteomics to unravel the community composition. The communities A, B, C, and D were distinct on phylum (Figure 6B and Supplementary Table S14) and family level (Supplementary Figure S9 and Supplementary Table S15). The phyla *Proteobacteria*, *Firmicutes* and *Bacteroides* dominated in all colonic communities, whereas the phylum *Actinobacteria* was low abundant. In contrast to the other communities, community B showed a high abundance of *Firmicutes* (Figure 6B). In line with this, the Bray-Curtis (BC) dissimilarity of community B was highest compared to the other communities based on the family abundances. Community A, C, and D showed a lower BC dissimilarity to each other than to community B (Figure 6C and Supplementary Table S16), proving community B to be different.

Riboflavin measurement unraveled that all colonic communities A, B, C, and D after blank subtraction had negative Δ riboflavin concentrations indicating riboflavin uptake from the culture medium (Figure 6D, *n* = 3, mean ± sd, one-way ANOVA, raw data: Supplementary Table S17 and Supplementary Figure S10). Of note, community B had the highest MAIT cell activating potential and used significantly less riboflavin than the communities A, C, and D (Figure 6D, *n* = 3, mean ± sd, GraphPad Prism, version: 8.3.0, one-way ANOVA, Tukey correction for multiple comparisons, *P* listed in Supplementary Table S18).

### In Vitro MAIT Cell Activation Is Inversely Related to Microbial Diversity

**FIGURE 7:**
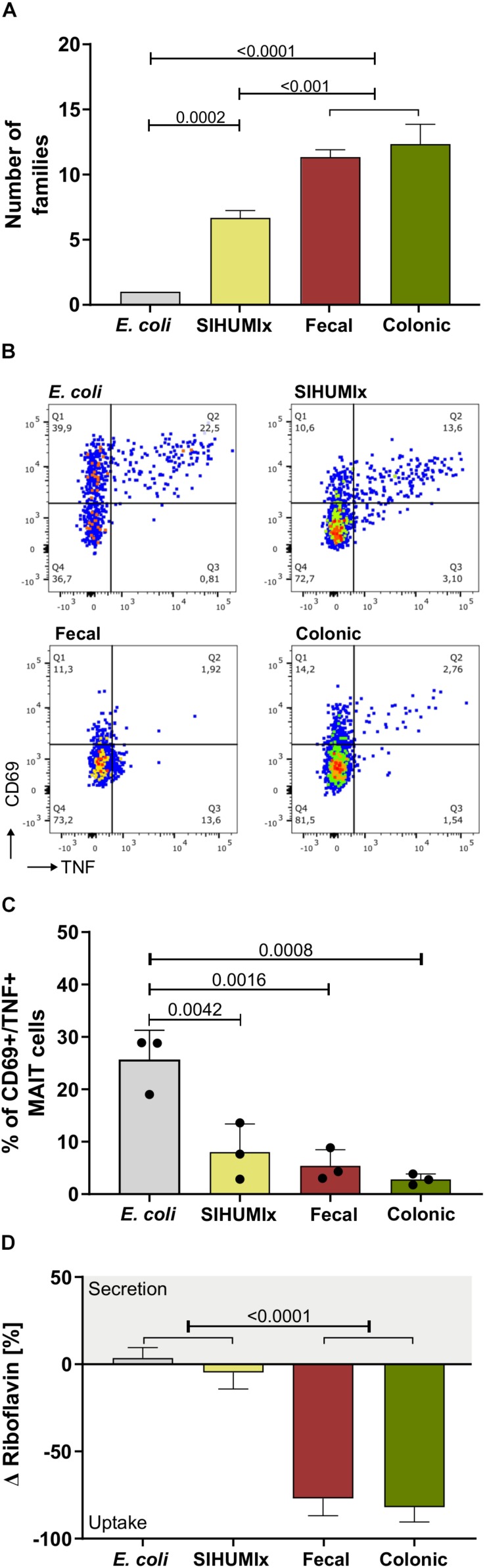
Comparing the MAIT cell activating potential with regard to microbial diversity. **(A)** We quantified microbial diversity by metaproteomics and therefore evaluated the number of families in human fecal communities,swine colonic communities, the SIHUMIx community and *E. coli.* **(B)** The exemplary dot plots show the percentage of CD69^+^/TNF^+^ activated MAIT cells after stimulation with fecal, colonic, SIHUMIx communities and *E. coli.* 200 bacteria per cell (BpC). **(C)** The bar plots quantify the percentage of CD69^+^/TNF^+^ activated MAIT cells after stimulation with fecal, colonic and SIHUMIx communities, and *E. coli*. Bars represent mean ± sd, *n* = 3, ordinary one-way ANOVA, Tukey *post hoc* test for multiple comparisons. **(D)** The riboflavin concentration was measured in the culture supernatant after microbiota cultivation [ng/mL]. Bars represent mean ± sd (*n* = 3). Positive changes indicate riboflavin secretion, negative values indicate riboflavin uptake.

Since a loss of microbial diversity and/or species richness in the intestine is associated with a variety of diseases (Mosca et al., 2016), we compared the MAIT cell response to microbial samples with different diversity. We used (i) diverse communities from cultivated human feces and swine colon content, (ii) the SIHUMIx model community composed of eight species, and (iii) the bacterial strain *E. coli*, a known MAIT cell activator. Microbial diversity was determined with two orthogonal methods: 16S rRNA gene analysis and microbial flow cytometry. For 16S rRNA gene analysis, the number of families were compared (Figure 7A, Supplementary Figure 11), whereas in microbial flow cytometry, the number of sub-populations in the corresponding cell gates were used to describe species richness (Supplementary Figure S12). Based on 16S rRNA sequencing, the fecal and the colonic communities both had the highest microbial diversity (number of families: 19 and 12, number of sub-populations: 21 and 31, respectively). The microbial diversity of SIHUMIx was significantly lower (number of families: 6, number of sub-populations: 19) and it was lowest for *E. coli* (number of families: 1, number of sub-populations: 5, representing different growth states, one-way ANOVA, *n* = 3, mean ± sd, *P* are listed in Supplementary Table S19). With regard to the MAIT cell activating potential, we observed the lowest potential to activate MAIT cells for the fecal and the colonic community (Figures 7B,C, *n* = 3, 5.4 and 2.8% CD69^+^/TNF^+^ MAIT cells, respectively). The SIHUMIx community had a slightly higher MAIT cell activating potential (8.0% CD69^+^/TNF^+^ MAIT cells), and *E. coli* showed the highest potential to activate MAIT cells (25.57% CD69^+^/TNF^+^ MAIT cells), respectively (one-way ANOVA, *n* = 3, mean ± sd, *P* are listed in Supplementary Table S20). The addition of anti-MR1 antibody significantly blocked the activation of MAIT cells. This suggests MR1-mediated antigen presentation (Supplementary Figure S13).

The microbiota were cultivated in different culture media. Therefore, we compared the riboflavin utilization with the Δ riboflavin concentration relative to the medium (Figure 7D). A negative Δ riboflavin value indicated riboflavin uptake, whereby a positive Δ riboflavin value suggested riboflavin secretion. For the fecal, the colonic and the SIHUMIx community, riboflavin was taken up from the medium. However, the fecal and the colonic community took up more riboflavin than SIHUMIx and *E. coli* (Figure 7E, one-way ANOVA, *n* = 3, mean ± sd, *P* are listed in Supplementary Table S21). For *E. coli* we observed a positive Δ riboflavin concentration, suggesting riboflavin biosynthesis and secretion. The riboflavin utilization of *E. coli* and SIHUMIx were similar but differed from the fecal and the colonic community. We observed that the riboflavin demand increased with microbial diversity.

## Discussion

In physiological context, MAIT cells probably interact with diverse microbiota, but the interaction of MAIT cells and diverse microbiota is still unexamined. Therefore, we have analyzed the response of MAIT cells to microbial communities. We used the extended simplified human microbiota (SIHUMIx) model community to investigate the contribution of individual community members to MAIT cell activation and investigated the MAIT cell response to diverse microbiota. Moreover, we analyzed whether acute microbial stress can indirectly affect MAIT cell activation.

MAIT cells recognize activating riboflavin metabolites and to a lower extent inhibitory folate metabolites after presentation on MR1. Thus, we speculated that riboflavin as well as folate concentrations secreted by individual bacteria strains might indicate the MAIT cell activating potential of the bacteria. The SIHUMIx bacteria cover the phyla *Bacteroides* (*B. thetaiotaomicron*), *Proteobacteria* (*E. coli*),*Actinobacteria* (*B. longum*), and *Firmicutes* (*A. caccae, B. producta, C. butyricum, C. ramosum*, and *L. plantarum*), for which has recently been shown that they activate MAIT cells in decreasing order (Tastan et al., 2018). In line with their results, *B. thetaiotaomicron* had the highest MAIT cell activating potential and at the same time secreted the highest amount of riboflavin into the medium. *E. coli* showed an intermediate and *L. plantarum* a low MAIT cell activating potential with simultaneously decreasing riboflavin secretion. For these MAIT cell activating bacterial strains, metaproteome data (proteome analysis and UniProt database) as well as genome data (KEGG database) proved the ability to synthesize riboflavin and thus the possible existence of MAIT cell activating metabolites (Figure 2 and Supplementary Table S11). On contrary, all strains unable to activate MAIT cells did not possess the full riboflavin biosynthesis pathway. The enzyme RibD/G (EC: 3.5.4.26/1.1.1.193) was reported in the two non-activating SIHUMIx strains *A. caccae* and *C. butyricum*. This enzyme has been identified as key enzyme for the synthesis of 5-A-RU (Kjer-Nielsen et al., 2018), however, pryp2 (EC: 3.1.3.104) is essential to perform the last enzymatic transformation toward 5-A-RU and is missing in the SIHUMIx strains *A. caccae* and *C. butyricum*.

A minority of MAIT cells can recognize folate metabolites (Gherardin et al., 2016), which are MAIT cell inhibitors *in vitro* (Kjer-Nielsen et al., 2012; Corbett et al., 2014). On contrary, the majority of TRAV1-2^+^ MAIT cells recognizes riboflavin metabolites via their TCR. Nevertheless, the activating riboflavin and the inhibitory folate metabolites compete for the MR1 binding site thereby impacting on MAIT cell activation independent of the TCR repertoire. *In vitro* studies show that the most potent inhibitory folate metabolite, Ac-6-FP, must exceed the molar concentration of 5-OP-RU, the strongest MAIT cell activator from the riboflavin pathway, by 10^6^ to inhibit the activating effect of 5-OP-RU (Corbett et al., 2014; Eckle et al., 2015). The folate concentration exceeded the riboflavin concentration in all the bacterial single strains from the SIHUMIx community. To determine the contribution of individual community members to the MAIT cell activating potential of a community, we stimulated MAIT cells with a SIHUMIx 1:1: mix. This SIHUMIx 1:1 mix contained all strains in equivalent numbers. We observed that the MAIT cell activating potential of the artificial SIHUMIx 1:1 mix equals the mean MAIT cell activating potential of the individual community members. Moreover, the folate/riboflavin ratio did not correlate with the MAIT cell activating potential of the SIHUMIx single strains (Supplementary Figure S3). It remains to be elucidated whether bacteria do not produce the inhibitory folate photo-degradation product 6-FP in culture e.g., due to darkness or whether 6-FP plays a minor role in MAIT cell activation. Indeed, we and others suppose a direct association between MAIT cell activation and riboflavin secretion on the single strain level at least under unimpeded conditions (Tastan et al., 2018).

Intestinal bacteria supply the host with a variety of B-vitamins and cross-feed nutrients with other species in the gut (LeBlanc et al., 2013; Magnusdottir et al., 2015; Rodionov et al., 2019). Genome analysis (2,228 genomes) unraveled that all bacteria from the phyla *Bacteroides* and *Proteobacteria* were prototrophic for riboflavin (Rodionov et al., 2019). Besides, bacteria from these phyla invariably were shown to activate MAIT cells in our study and others (Tastan et al., 2018). 60% of all *Firmicutes* and *Actinobacteria* in the study of Rodionov et al. (2019) were unable to synthesize riboflavin and should therefore be unable to activate MAIT cells, which is in line with our data. Nevertheless, riboflavin is essential for all organisms to synthesize flavin mononucleotide (FMN) and flavin adenine dinucleotide (FAD) and thus has to be taken up (Gutiérrez-Preciado et al., 2015; García-Angulo, 2017). Accordingly, the reduced riboflavin concentration in the culture supernatant of the non-MAIT cell activating bacterial strains *A. caccae, B. longum, B. producta, C. butyricum*, and *C. ramosum* suggests riboflavin uptake and thereby riboflavin auxotrophy. We show that the non-activating SIHUMIx strains lack the riboflavin biosynthesis pathway completely or in parts and in parallel possess the enzymatic equipment for riboflavin uptake and conversion to FAD and FMN. Riboflavin biosynthesis is strongly dependent on extracellular riboflavin and the individual demand of microbial species (Gutiérrez-Preciado et al., 2015). Furthermore, extracellular riboflavin favors riboflavin import and in parallel inhibits riboflavin biosynthesis (García-Angulo, 2017), which presumably bypasses the synthesis of MAIT cell activating metabolites. We suggest that the MAIT cell activating potential of individual species, but also microbial communities, depends on the availability of riboflavin in the culture medium and on the culture conditions, which together determine the need for riboflavin biosynthesis. In support of this, culture conditions have recently been identified to alter the MAIT cell activating potential of *E. coli* (Schmaler et al., 2018).

Opposed to the situation in single strains, all microbial communities (fecal, colonic, SIHUMIx) cultivated in our study removed riboflavin from the culture medium and still had the potential to activate MAIT cells. However, the MAIT cell activating potential decreased with increasing community diversity. Especially in symbiotic or commensal interactions, like those found in the intestine, secretion of metabolites such as riboflavin are important for symbiosis establishment, maintenance and microbial cross-feeding (LeBlanc et al., 2013; Rowland et al., 2018; Rodionov et al., 2019). Within communities, the expression of enzymes from the riboflavin biosynthesis pathway (FMN riboswitch) is regulated in various ways. E.g., riboflavin can suppress the expression of genes coding for riboflavin biosynthesis enzymes to maintain an energy efficient riboflavin biosynthesis. It is suggested, that the majority of riboflavin is used by bacteria that do not actively produce the vitamin (Gutiérrez-Preciado et al., 2015; Rowland et al., 2018). Thus, in diverse communities, we suggest that some prototrophic bacteria supply riboflavin and possess MAIT cell activating metabolites, whereas many other bacteria take up riboflavin and do not produce MAIT cell activating metabolites.

To investigate if microbial stress can affect the MAIT cell activation downstream, we exposed the model community SIHUMIx to an acute acid stress. One major advantage of the SIHUMIx model community is the reproducible development and quick adaptation to the bioreactor system (Krause et al., 2020). During the adaptation phase to the bioreactor system, the Δ riboflavin concentration leveled around zero, which suggests an even riboflavin balance and accounts for riboflavin cross-feeding in an energy efficient manner (LeBlanc et al., 2013; Magnusdottir et al., 2015). Under acute acid stress, the SIHUMIx community lost its MAIT cell activating potential. However, we did not observe changes in community composition, but the communities’ riboflavin demand increased. Adaptation to stress shifts energy and nutrient flows, since microbial function or survival are threatened. Shifting from growth to survival-related metabolism sustains survival under acute stress, whereas changes in community composition are thought to occur as a response to long-term stress (Schimel et al., 2007). In *E. coli* flavoenzymes are involved in a variety of processes, e.g., oxidative stress response, which is directly associated with acid stress and presumably survival-related metabolism (Maurer et al., 2005; García-Angulo, 2017). FMN and FAD are used for the synthesis of flavoenzymes, which make up to 2% of coded genes (Gutiérrez-Preciado et al., 2015). The SIHUMIx strains possess riboflavin transporter and enzymes for riboflavin conversion to flavin cofactors (FMN and FAD), irrespective of their ability to synthesize riboflavin themselves (Supplementary Table S11). We propose that acid stress non-specifically affected the SIHUMIx community and stimulated riboflavin uptake for the synthesis of flavoenzymes in order to survive. Furthermore, we assume that the prototrophic SIHUMIx strains (*B. thetaiotaomicron, E. coli*, and *L. plantarum*) directly utilized their MAIT cell activating metabolites for riboflavin synthesis or completely stopped riboflavin biosynthesis in order to use GTP for survival. Either way, the prototrophic strains lose their MAIT cell activating potential during the stress response. The exact mechanism of riboflavin biosynthesis under stress should be investigated in the future. In addition, the riboflavin auxotrophic bacterial strains had an elevated demand for riboflavin and thus increased riboflavin uptake from the medium to synthesize flavoenzymes, which became visible in the decreased riboflavin concentration. After the acute acid stress ended, the MAIT cell activating potential of SIHUMIx as well as the riboflavin concentration recovered. Since the availability of riboflavin precursors was affected under acute acid stress, we hypothesize that riboflavin can serve as a microbial stress sensor, which mediates microbial stress to MAIT cells at least under acute stress conditions. IBD patients indeed can have a low colonic pH (Nugent et al., 2001), but here blood MAIT cells were chronically activated (Serriari et al., 2014). In contrast to IBD, in the bioreactors we introduced an acute stress for 24 h, whereas in IBD the gut is chronically inflamed.

With regard to diverse microbial communities, we observed that the most potent colonic community with regard to MAIT cell activation at the same time had the lowest riboflavin demand. In this community, the phylum *Firmicutes* dominated with ∼80% phyla abundance over the other phyla. In conflict with our result, Tastan et al. designated bacteria from the phylum *Firmicutes* low or no MAIT cell stimulators (Tastan et al., 2018). However, genome analysis predicted a functional riboflavin biosynthesis pathway to ∼50% of analyzed *Firmicutes*, indicating at least a 50% potential to activate MAIT cells (Gutiérrez-Preciado et al., 2015; Magnusdottir et al., 2015; Rodionov et al., 2019). Taxonomic analysis alone was insufficient to estimate the MAIT cell activating potential of the communities. However, concluding from our results riboflavin utilization seems to be a good approximation for the MAIT cell activating potential of microbial communities *in vitro*.

Especially the MAIT cell activating potential of bacterial single strains is directly related to the riboflavin secretion. Our data indicate that the MAIT cell activating potential of microbial communities on the one hand correlated with the riboflavin demand and on the other hand with microbial diversity. A high microbial diversity was associated with a higher demand of riboflavin combined with a low MAIT cell activating potential. Furthermore, we show that acute microbial stress can indirectly affect the MAIT cell activation downstream *in vitro* via riboflavin metabolism. Therefore, we hypothesize that microbial stress can be mediated to MAIT cells via riboflavin utilization.

## Data Availability Statement

The raw data supporting the conclusions of this article will be made available by the authors, without undue reservation, to any qualified researcher.

## Author Contributions Statement

JK and GH conceptualized the study. JK cultivated the bacteria in bioreactors, wrote the manuscript’s first draft, prepared the single strain bacteria and performed the immunologic experiments and prepared the samples for microbial flow cytometry. UR-K was responsible for riboflavin analysis. GH and GA contributed to the folate analysis. SS accomplished metaproteome analysis and together with NJ analyzed the data. FS performed flow cytometric measurement of all microbial samples and together with SM evaluated the data. GH, AP, SM, and MB provided helpful discussions and revised the manuscript.

## Conflict of Interest

GA was employed by Alphaomega laboratory. The remaining authors declare that the research was conducted in the absence of any commercial or financial relationships that could be construed as a potential conflict of interest.

## Footnotes

1 http://www.uniprot.org/
