# Supplementary material for: The Activation of Mucosal-Associated Invariant T (MAIT) Cells Is Affected by Microbial Diversity and Riboflavin Utilization in vitro
Source: Front Microbiol. 2020 Apr 22;11:755. doi: 10.3389/fmicb.2020.00755 (PMC7189812; doi:10.3389/fmicb.2020.00755)
Supplement: Supplementary file 1 [file Data_Sheet_1.PDF]

Supporting information

The activation of mucosal-associated invariant T (MAIT) cells is affected by microbial diversity and riboflavin utilization *in vitro*

JL Krause[1], SS Schaepe[2], F Schattenberg[3], S Mueller[3], G Ackermann[5], U Rolle-Kampczyk[2], N Jehmlich[2], A Pierzchalski[1], M von Bergen[2, 4], G Herberth[1]

[1] Helmholtz-Centre for Environmental Research - UFZ, Department of Environmental Immunology, Leipzig, Germany

[2] Helmholtz-Centre for Environmental Research - UFZ, Department of Molecular Systems Biology, Leipzig, Germany

[3] Helmholtz-Centre for Environmental Research - UFZ, Department of Environmental Microbiology, Leipzig, Germany

[4] Institute of Biochemistry, Faculty of Biosciences, Pharmacy and Psychology, University of Leipzig, Germany

Summary

Number of pages: 34

Number of Supporting Figures: 13

Number of Supporting Tables: 21

Table S 1. Supplemented Brain-Heart-Infusion medium (BHI). Storage at 4 °C

**Ingredient**	**Quantity [g or mL/L]**	**Supplier**
Brain-Heart-Infusion	37	Roth
L-cysteine hydrochloride	0.5	Biochemica
Resazurin	0.001	MP biomedicals
Vitamin K hemin solution	10	Becton Dickinson
Yeast extract	5	Chemsolute

Table S 2. Complex intestinal medium (CIM). PH was adjusted to pH 6.7 with NaOH. Storage at 4 °C.

**Ingredient**	**Quantity [g/L]**	**Supplier**
Arabinogalactan (larch wood)	2	Sigma-Aldrich
Bile Acids sodium salt	0.5	Sigma-Aldrich
Calcium chloride x 2 H_2O	0.01	Merck
Casein peptone (pancreatic)	4.3	Roth
Di-Potassium hydrogen phosphate	0.04	Roth
Hemin (bovine)	0.005	Sigma-Aldrich
Inulin	1	Serva
L-cysteine hydrochloride	0.5	Biochemica
Magnesium sulfate	0.01	Roth
Menadione	0.001	Sigma-Aldrich
Mucin (porcine gastric Type II)	4	Sigma-Aldrich
Pectin, citrus peel	2	Sigma-Aldrich
Potassium di-hydrogen phosphate	0.04	Roth
Sodium chloride	0.72	Roth
Sodium hydrogen carbonate	2	Roth
Starch, wheat	5	Roth
Xylo-oligosaccharide (corn)	2	Roth
Yeast extract	2	Chemsolut

Table S 3. Complex intestinal medium (CIM) adjusted to swine. Medium pH was adjusted to pH 6.7 with NaOH. Storage at 4 °C.

**Ingredient**	**Quantity [g/L]**	**Supplier**
Arabinogalactan (larch wood)	2	Sigma-Aldrich
Bile Acids sodium salt	0.5	Sigma-Aldrich
Calcium chloride x 2 H_2O	0.15	Merck
soy peptone, papainic	13	Roth
Di-Potassium hydrogen phosphate	0.25	Roth
Guar gum	2	Sigma-Aldrich
Hemin, bovine	0.05	Sigma-Aldrich
Inulin	1	Serva
L-cysteine hydrochloride	0.5	Biochemica
Magnesium sulfate	0.64	Roth
Menadione	0.001	Sigma-Aldrich
Mucin, porcine gastric Type II	4	Sigma-Aldrich
Pectin, citrus peel	2	Sigma-Aldrich
Potassium di-hydrogen phosphate	0.25	Roth
Sodium chloride	4.5	Roth
Sodium hydrogen carbonate	1.5	Roth
Starch, maize	4.21	Roth
Xylo-oligosaccharide, corn	2	Roth
Yeast extract	4.5	Chemsolut

Table S 4. List of antibodies for MAIT cell cytometric analysis. Antibodies were obtained from Biolegend and eBioscience.

Antibody	Fluorophor	Clone	Concentration	Supplier
Anti-CD3	FITC	UCHT1	1:100	Biolegend
Anti-CD8α	APC eF780	RPA-T8	1:200	eBioscience
Anti-CD161	BV421	HP-3G10	1:100	Biolegend
Anti-TCRv7.2	PerCP-Cy5.5	3C10	1:100	Biolegend
Anti-CD69	APC	FN50	1:200	Biolegend
Anti-TNF	PE	MAb11	1:400	Biolegend
Anti-IFNγ	PE/Cy7	B27	1:400	Biolegend
LEAF Anti-MR1	none		1:250	Biolegend
Fixable Viability Dye eFluor™ 506			1:500	eBioscience

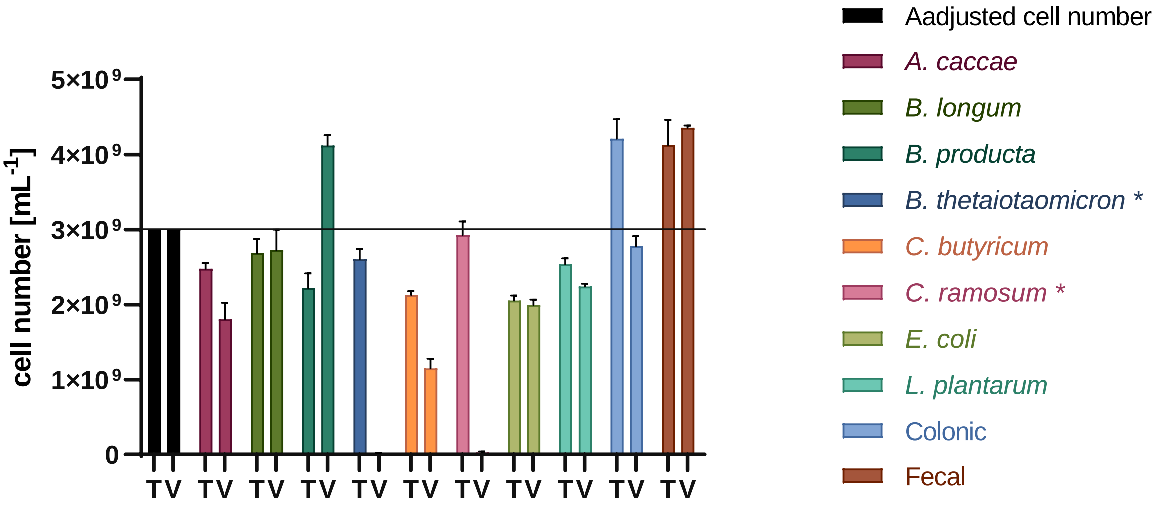

Figure S 1. Quantification of bacteria from frozen bacteria pellets. Bacteria pellets (cell number 3 x 10^9 cells/mL) were thawed and cells were stained to determine the total (T) and the viable (V) cell number at the QuantomTX Microbial Cell Counter. The number of total cells (T) was decreased, alike the number of viable cells (V, n=3, mean ± sd) indicating a loss of intact bacteria presumably via lysis. However, the cells mostly remained intact and to a great extant viable. The viable cell number of *B. thetaiotaomicron* and *C. ramosum* could not be determined. Exemplary images: Figure S2.

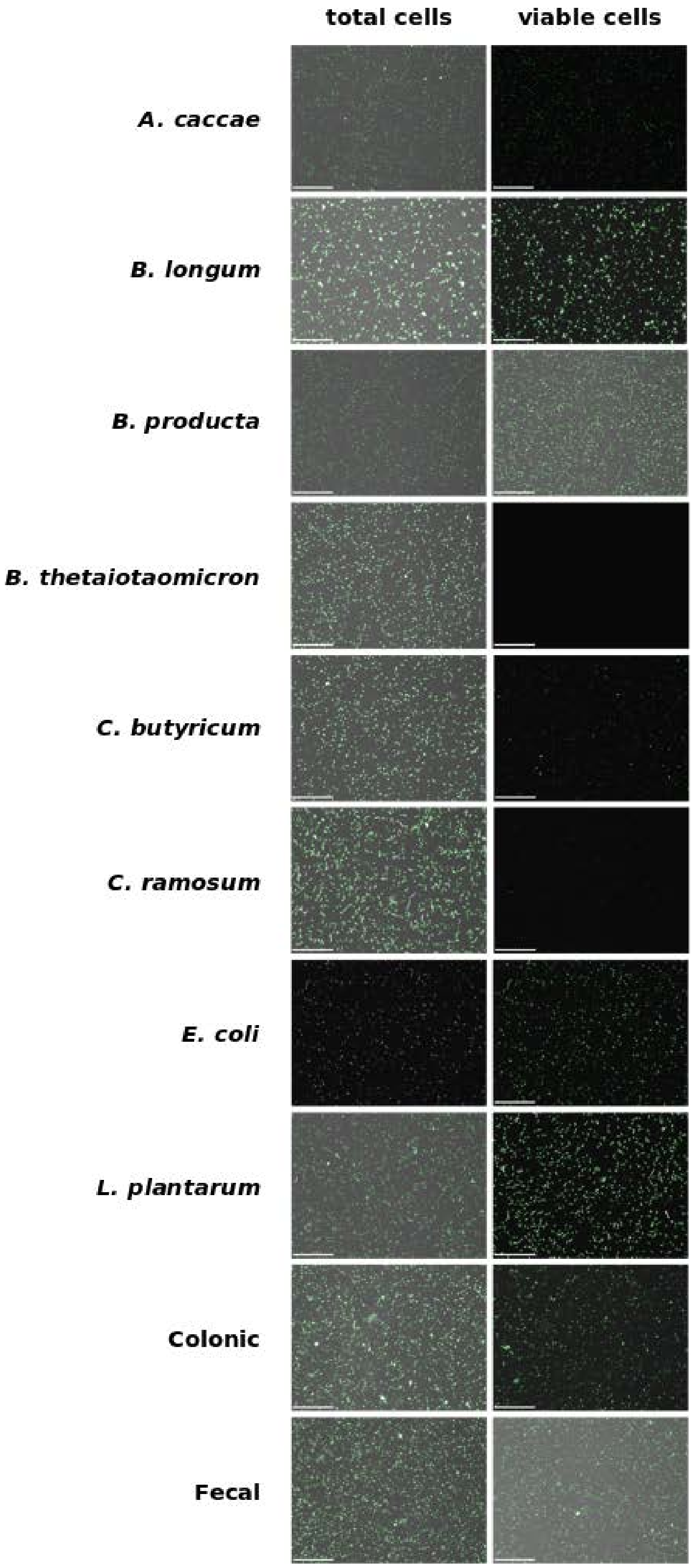

Figure S 2. Staining images from the QuantomTX Microbial Cell counter. The total cell number and the viable cell number were determined. Therefore, cells were stained according to the manufacturers' instructions. The results are summarized in Figure S1.

Table S 5. Riboflavin analysis concentration in [ng/mL]. The SIHUMIx single strains were cultivated for 24 h in Brain-Heart-Infusion (BHI) medium. Afterwards the bacteria were pelleted by centrifugation and riboflavin [ng/mL] was measured in the culture supernatant.

Bacterial strain	Replicate 1	Replicate 2	Replicate 3
A. caccae	23,9	31,3	29,8
B. longum	75,3	81,7	81,0
B. producta	45,2	54,9	54,7
B. thetaiotaomicron	88,0	97,8	95,5
C. butyricum	59,9	71,3	78,6
C. ramosum	75,6	79,5	74,2
E. coli K12	85,6	96,1	92,5
L. plantarum	71,6	99,1	97,3
BHI	109,0	74,6	81,3

Table S 6. Raw data for the targeted folate analysis – concentration in [ng/mL]. The SIHUMIx single strains were cultivated for 24 h in Brain-Heart-Infusion (BHI) medium. Afterwards the bacteria were remove by centrifugation and total folate [ng/mL] was measured in the culture supernatant.

Bacterial strain	Replicate 1	Replicate 2	Replicate 3
A. caccae	508	615	456
B. longum	1133	826	778
B. producta	1227	1353	1250
B. thetaiotaomicron	1459	1096	1023
C. butyricum	1146	1099	809
C. ramosum	na	842	1009
E. coli K12	1343	1072	592
L. plantarum	1347	956	1200
BHI	346	516	601

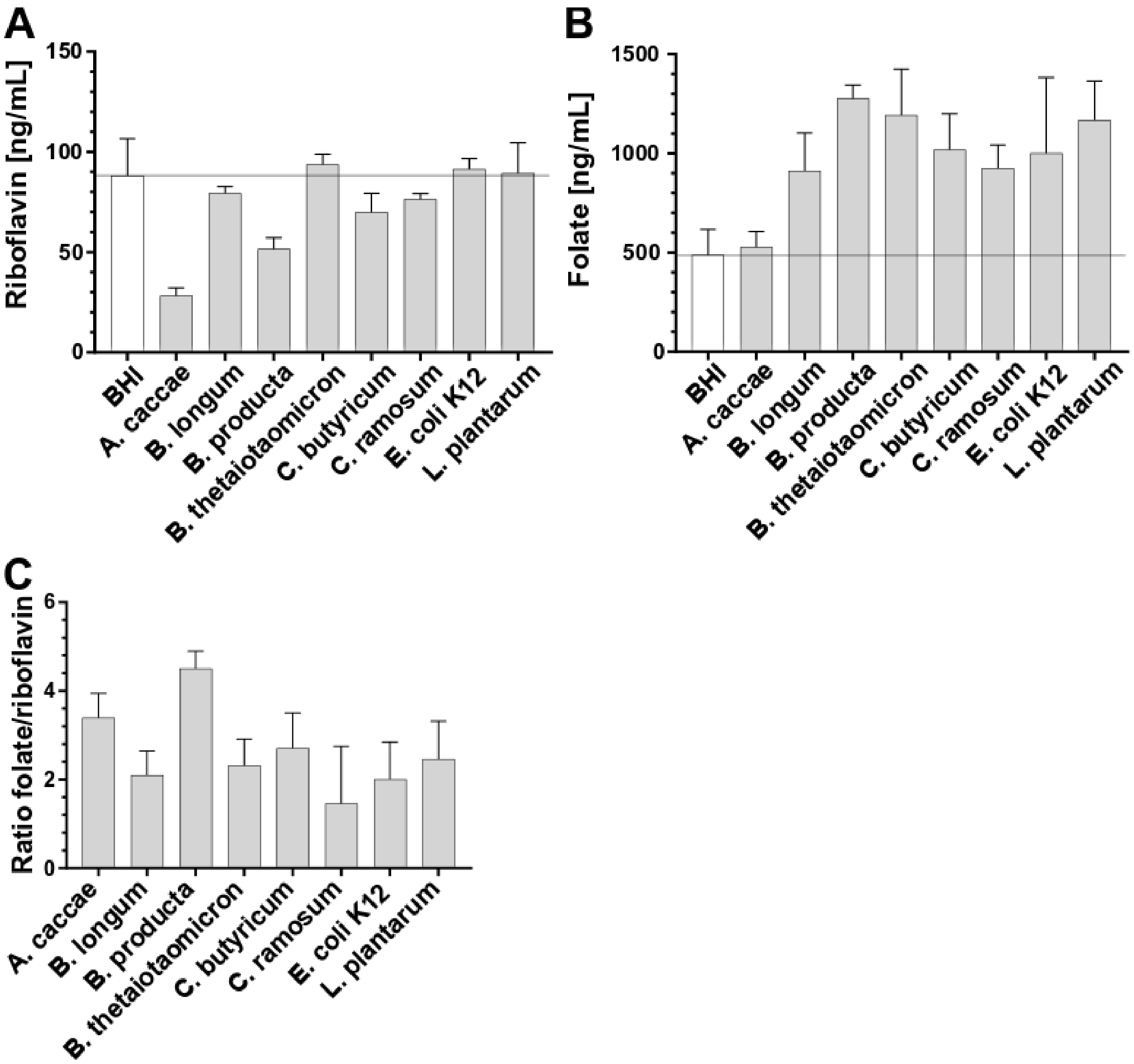

Figure S 3. Raw data for riboflavin and folate quantitation – absolute concentration in [ng/mL]. The SIHUMIx single strains were cultivated for 24 h in Brain-Heart-Infusion (BHI) medium. Afterwards the bacteria were remove by centrifugation and **(A)** riboflavin and **(B)** total folate were measured in the culture supernatant. **(C)** The folate/riboflavin ratio was calculated with the medium background set to 100.

Table S 7. Riboflavin biosynthesis pathway analysis of SIHUMIx bacterial strains. Detected enzymes are marked with U (UniProt database), K (KEGG database) or P (proteomics analysis) when detected. The grey background marks enzymes that follow riboflavin biosynthesis in the riboflavin pathway. The following proteomes were used in UniProt (06.11.2019): *A. caccae* - UP000004935, *B. longum* - UP000000439, *B. producta* – UP000236922 (closest related genome), *B. thetaiotaomicron* – UP000001414, *C. butyricum* – UP000003081 (closest related genome), *C. ramosum* – UP000005798, *E. coli* K12 – UP000000625, *L. plantarum* – UP000005567. For KEGG database search the following strains were used (11.02.2020): *B. longum* NCC2705, *B. producta* PMF1, *B. thetaiotaomicron* VPI-5482 & 7330, *C. butyricum* KNU-L09, *L. plantarum* WCFS-1, *E. coli* K12 MG1655.

EC numbers	Enzyme name	A.c.	B.l	B.p.	B.t.	C.b.	C.r.	E.c.	L.p.
**3.5.4.25**	**ribA**	U			U K P	U K		U K P	U K P
**4.1.99.12**	**ribB**	U			U K P	U K		U K P	K P
**3.5.4.26/1.1.1.193**	**ribD**	U			U K P	U K		U K P	U
**3.1.3.104**	**pyrp2**				U K			U K	K
**2.5.1.78**	**ribH**				U K P	U K		U K P	U K
**2.5.1.9**	**ribE**	U			K P	U K		U K P	U
**2.7.1.26**		U P	K	U K	U K P	U K	U	U K P	U K
**2.7.7.2**		U P	K	U K	U K P	U K	U	U K P	U K
**Riboflavin transporter**		U	U	U		U			U

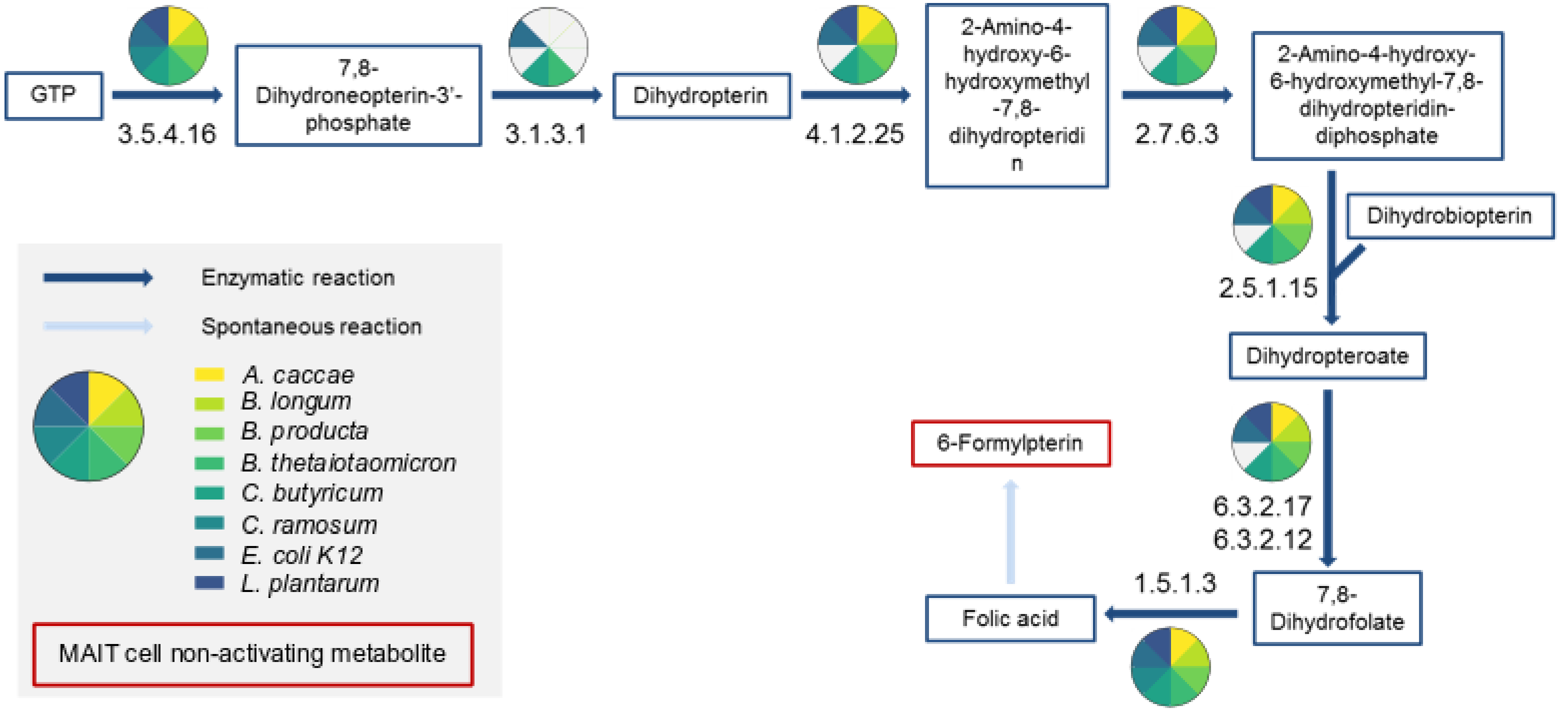

Figure S 4. Folate biosynthesis pathway analysis of SIHUMIx bacterial strains. Enzymes from the folate biosynthesis pathway are coded by their EC number. The colors used in the pie charts indicate detected proteins of corresponding bacterial strains. Metabolites that can be recognized by MAIT cells after presentation on MHC I related protein 1 (MR1) are marked in red

Table S 8. Folate biosynthesis pathway analysis of SIHUMIx bacterial strains. Detected enzymes are marked with U (UniProt database), K (KEGG database) or P (proteomics analysis) when detected. The following proteomes were used in UniProt (06.11.2019): *A. caccae* - UP000004935, *B. longum* - UP000000439, *B. producta* – UP000236922 (closest related genome), *B. thetaiotaomicron* – UP000001414, *C. butyricum* – UP000003081 (closest related genome), *C. ramosum* – UP000005798, *E. coli* K12 – UP000000625, *L. plantarum* – UP000005567. For KEGG database search the following strains were used (11.02.2020): *B. longum* NCC2705, *B. producta* PMF1, *B. thetaiotaomicron* VPI-5482 & 7330, *C. butyricum* KNU-L09, *L. plantarum* WCFS-1, *E. coli* K12 MG1655.

**EC numbers**	**A.c.**	**B.l**	**B.p.**	**B.t.**	**C.b.**	**C.r.**	**E.c.**	**L.p.**
**3.5.4.16**	U	U K	U K P	U K P	U K P	U	U K P	U K
**3.1.3.1**				K	K		U K	
**4.1.2.25**	U P	U K	U K P	K	U K		U K	U K
**2.7.6.3**	U P	K	K P	K	K		U K	U K
**2.5.1.15**	U P	U K	U K P	K P	U K		U K	U K
**6.3.2.17/6.3.2.12**	U P	K	K	K P	U K		U K	U K
**1.5.1.3**	U P	U K	U K	U K P	U K	U	U K	U K

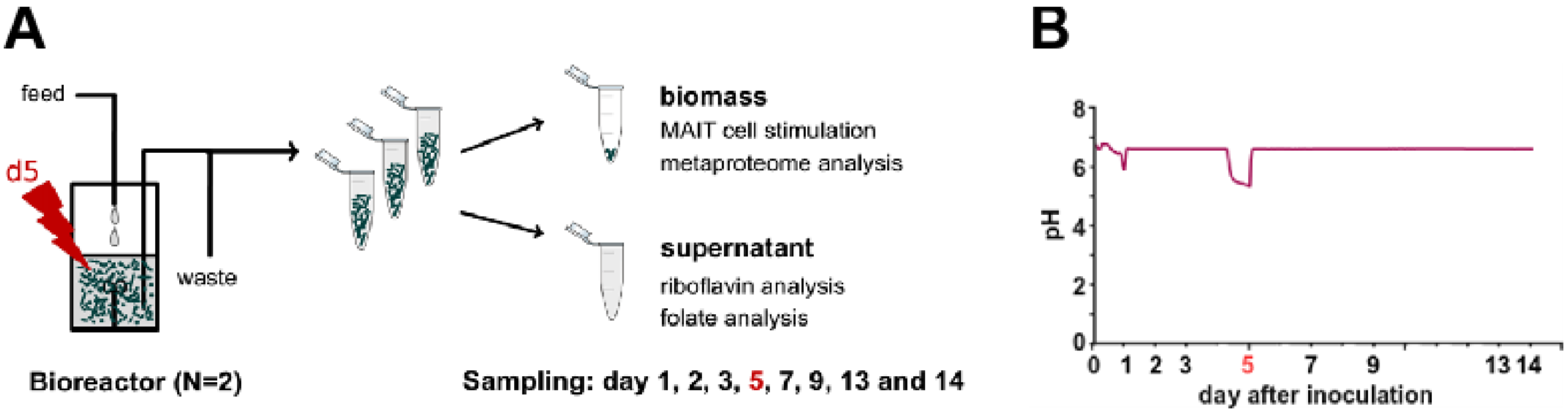

Figure S 5. Experimental setup for the cultivation of SIHUMIx in order to investigate both, the effect of unstressed and acid stressed culture conditions on the MAIT cell activating potential of SIHUMix. **(A)** The SIHUMIx community (n=2 per condition) was cultivated *in vitro*. Four replicate bioreactors were inoculated (day 0) and after 24 h continuous cultivation started. On day 4, the pH was set from 6.5 to 5.5 to induce a microbial acid stress for 24 h in two bioreactors, whereas the other two bioreactors remained untreated. After sampling on day 5, the pH was reset to 6.5. Samples were taken on day 1, 2, 3, 5, 7, 9, 13 and 14 for MAIT cell stimulation, metaproteomics and riboflavin and folate analysis. **(B)** During the bioreactor experiment the pH was monitored permanently. The pH on day 4 was reduced from 6.5 to 5.5 for 24 h in the stressed bioreactors.

Table S 9. Comparison of the MAIT cell response after stimulation with SIHUMIx communities. The SIHUMIx communities from the bioreactor were used for MAIT cell stimulation. On day 5, the SIHUMIx communities were set under acid by a pH reduction from 6.5 to 5.5. The potential to activate MAIT cells (equal to the number of $CD69^{+}/TNF^{+}$ MAIT cells) was compared using one-way ANOVA (GraphPad Prism, version: 8.3.0) with Tukey correction for multiple comparisons.

**Sample**	**Significance**	***P***
d1 vs. d2	ns	>0.9999
d1 vs. d3	*	0.0391
d1 vs. d5	***	0.0008
d1 vs. d7	**	0.0032
d1 vs. d9	ns	0.3789
d1 vs. d12	ns	0.9901
d1 vs. d14	ns	>0.9999
d2 vs. d3	*	0.0252
d2 vs. d5	***	0.0005
d2 vs. d7	**	0.002
d2 vs. d9	ns	0.2739
d2 vs. d12	ns	0.9987
d2 vs. d14	ns	0.998
d3 vs. d5	ns	0.4916
d3 vs. d7	ns	0.8886
d3 vs. d9	ns	0.866
d3 vs. d12	**	0.0082
d3 vs. d14	ns	0.0808
d5 vs. d7	ns	0.9941
d5 vs. d9	ns	0.0579
d5 vs. d12	***	0.0002
d5 vs. d14	**	0.0016
d7 vs. d9	ns	0.2099
d7 vs. d12	***	0.0007
d7 vs. d14	**	0.0068
d9 vs. d12	ns	0.1061
d9 vs. d14	ns	0.5982
d12 vs. d14	ns	0.9212

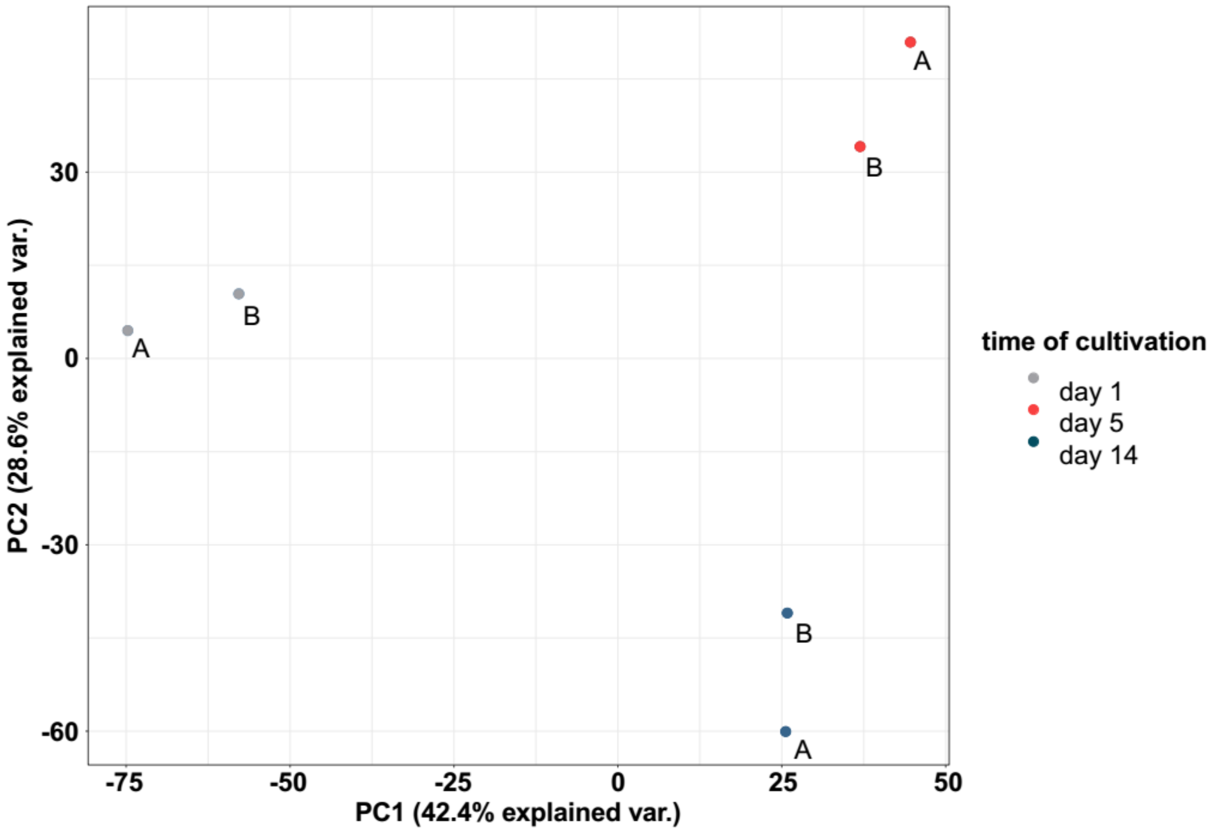

Figure S 6. PCA analysis based on relative species abundances shows differences between the communities on day 1, day 5 and day 14. The model community SIHUMIx was cultivated in duplicate bioreactors (A and B). The initial SIHUMIx community (day 1), the community under acid stress (day 5) and the recovered community (day 14) segregate are different.

Table S 10. Relative species abundances of SIHUMIx on day 1, day 5 and day 14 based on shotgun metaproteomics. The SIHUMIx communities were cultivated in duplicate bioreactors A and B. Shotgun metaproteome analysis were performed on day 1, day 5 and day 14 from the bacteria biomass for both bioreactors. Mean relative species abundances were shown as stacked bar plot in Figure S5 (A) and original data from bioreactor A and B were used for PCA analysis Figure S5.

Bacterial strain	A d1	A d5	A d14	B d1	B d5	B d14	Mean d1	Mean d5	Mean d14
Anaerostipes caccae	6.10	8.14	4.59	6.32	8.78	4.62	6.21	8.46	4.60
Bacteroides thetaiotaomicron	71.77	57.12	69.59	72.04	62.96	71.95	71.90	60.04	70.77
Bifidobacterium longum	0.00	0.01	0.00	0.00	0.00	0.00	0.00	0.00	0.00
Blautia sp.	7.88	20.86	16.46	8.19	18.55	16.43	8.04	19.70	16.44
Clostridium butyricum	0.00	0.00	0.00	0.00	0.00	0.00	0.00	0.00	0.00
Clostridium ramosum	0.01	0.11	0.04	0.01	0.08	0.04	0.01	0.10	0.04
Escherichia coli	14.19	13.50	9.29	13.40	9.45	6.94	13.79	11.48	8.11
Lactobacillus plantarum	0.04	0.27	0.03	0.04	0.18	0.03	0.04	0.22	0.03

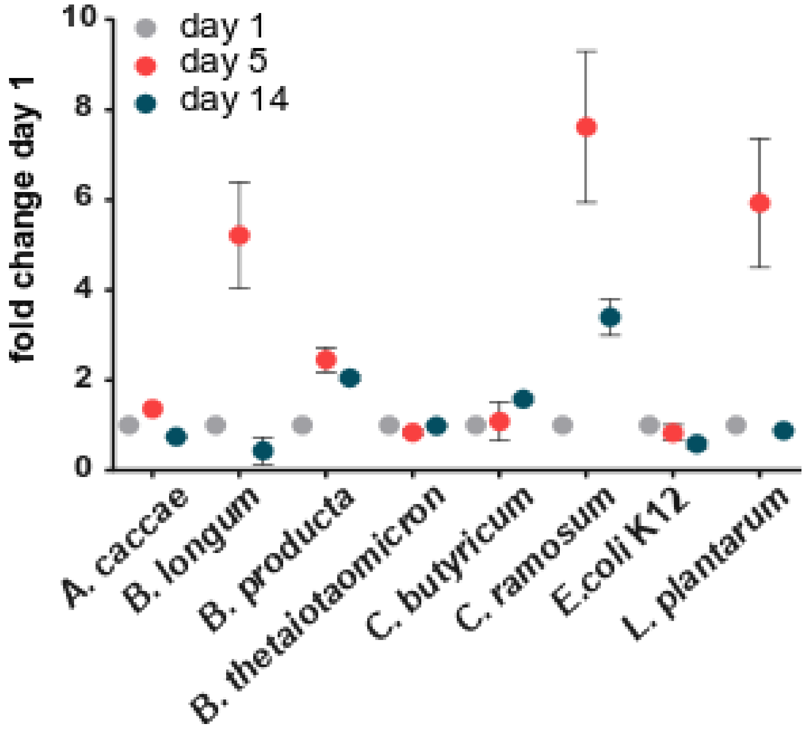

Figure S 7. The relative species abundances of SIHUMIx are similar, but differ in the low abundant strains. The relative species abundances are plotted as fold change of day 1 for day 1, day 5 and day 14.

Table S 11. Mean values of the riboflavin and folate concentration in the culture supernatant of SIHUMIx.

	**CIM**	**d1**	**d2**	**d3**	**d5**	**d7**	**d9**	**d13**	**d14**
**Riboflavin**	163	257.5	251.17	166.5	108.5	31	46.2	168	142.7
**Folate**	154	909	2019	1675.5	1461	1495.5	1680	1848	1791
**Ribo/Folate**	0.94	3.53	8.04	10.06	13.47	48.24	36.35	11.00	12.55

Table S 12. Comparison of riboflavin concentrations after blank subtraction**.** Riboflavin was measured in the supernatant of cultivated SIHUMIx communities. On day 5, the SIHUMIx communities were exposed to an acid stress by a pH reduction from 6.5 to 5.5 for 24 h. The riboflavin concentrations were compared using one-way ANOVA (GraphPad Prism, version: 8.3.0) with Tukey correction for multiple comparisons.

**Sample**	**Significance**	***P***
d1 vs. d2	ns	0.9791
d1 vs. d3	****	<0.0001
d1 vs. d5	****	<0.0001
d1 vs. d7	****	<0.0001
d1 vs. d9	****	<0.0001
d1 vs. d12	****	<0.0001
d1 vs. d14	****	<0.0001
d2 vs. d3	****	<0.0001
d2 vs. d5	****	<0.0001
d2 vs. d7	****	<0.0001
d2 vs. d9	****	<0.0001
d2 vs. d12	****	<0.0001
d2 vs. d14	****	<0.0001
d3 vs. d5	****	<0.0001
d3 vs. d7	****	<0.0001
d3 vs. d9	****	<0.0001
d3 vs. d12	ns	>0.9999
d3 vs. d14	*	0.0196
d5 vs. d7	****	<0.0001
d5 vs. d9	****	<0.0001
d5 vs. d12	****	<0.0001
d5 vs. d14	***	0.0002
d7 vs. d9	ns	0.3318
d7 vs. d12	****	<0.0001
d7 vs. d14	****	<0.0001
d9 vs. d12	****	<0.0001
d9 vs. d14	****	<0.0001
d12 vs. d14	*	0.0107

Table S 13. Comparison of folate concentrations after blank subtraction. Folate was measured in the supernatant of cultivated SIHUMIx communities. On day 5, the SIHUMIx communities were exposed to an acid stress by a pH reduction from 6.5 to 5.5 for 24 h. The folate concentrations were compared using one-way ANOVA (GraphPad Prism, version: 8.3.0) with Tukey correction for multiple comparisons.

Sample	Significance	*P*
d1 vs. d2	ns	0.0711
d1 vs. d3	ns	0.2968
d1 vs. d5	ns	0.6205
d1 vs. d7	ns	0.5606
d1 vs. d9	ns	0.2916
d1 vs. d12	ns	0.1468
d1 vs. d14	ns	0.1862
d2 vs. d3	ns	0.9294
d2 vs. d5	ns	0.6100
d2 vs. d7	ns	0.6704
d2 vs. d9	ns	0.9334
d2 vs. d12	ns	0.9984
d2 vs. d14	ns	0.9914
d3 vs. d5	ns	0.9939
d3 vs. d7	ns	0.9979
d3 vs. d9	ns	>0.9999
d3 vs. d12	ns	0.9984
d3 vs. d14	ns	0.9999
d5 vs. d7	ns	>0.9999
d5 vs. d9	ns	0.9931
d5 vs. d12	ns	0.8826
d5 vs. d14	ns	0.9411
d7 vs. d9	ns	0.9975
d7 vs. d12	ns	0.9208
d7 vs. d14	ns	0.9654
d9 vs. d12	ns	0.9986
d9 vs. d14	ns	>0.9999
d12 vs. d14	ns	>0.9999

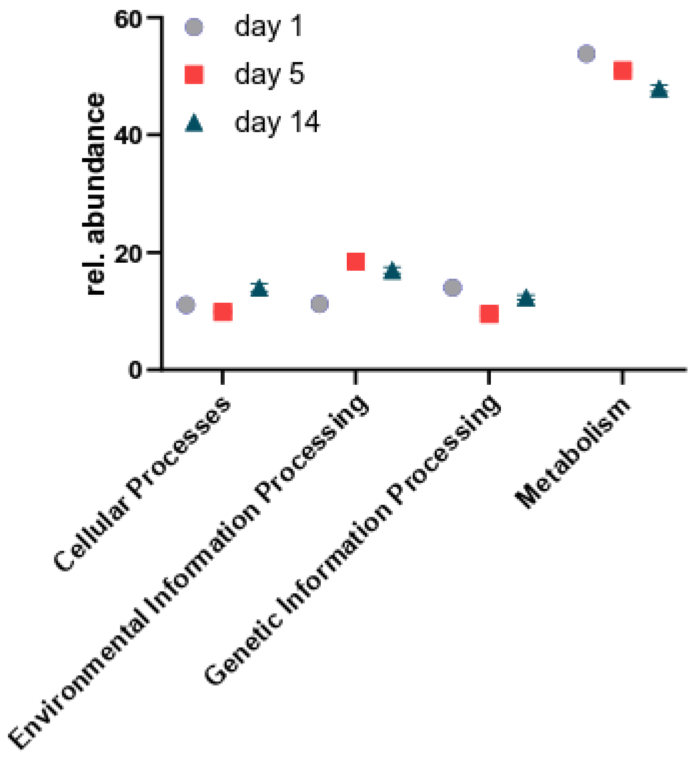

Figure S 8. Relative protein abundances in KEGG categories related to metabolism. The KEGG categories *cellular processes, metabolism, environmental information processing* and *genetic information processing* were similar on day 1, day 5 and day 14 independent of the acid stress.

Table S 14. Relative phyla abundances in the complex colonic communities A-D on day 21 based on shotgun metaproteomics.

Phylum	Community A	Community B	Community C	Community D
Actinobacteria	1.38	1.14	1.53	0.90
Bacteroidetes	22.87	1.92	32.91	40.52
Firmicutes	48.13	79.56	31.13	43.39
Proteobacteria	27.62	17.38	34.43	15.19

Table S 15. Relative family abundances in the complex colonic communities A-D on day 21 based on shotgun metaproteomics.

Family	Community A	Community B	Community C	Community D
Enterobacteriaceae	20.44	21.89	19.94	21.67
Ruminococcaceae	6.76	13.48	7.25	8.44
Bacteroidaceae	8.71	6.25	6.98	7.52
Lachnospiraceae	9.22	10.67	7.38	8.11
Bifidobacteriaceae	3.65	3.54	4.39	4.06
Lactobacillaceae	8.10	5.45	7.07	5.79
Prevotellaceae	5.76	4.75	5.16	6.92
Clostridiaceae	3.31	4.09	3.27	4.52
Peptococcaceae	3.67	2.57	4.21	1.90
Yersiniaceae	3.37	2.24	3.11	4.02
Morganellaceae	1.78	1.35	3.92	2.86
Rhodospirillaceae	2.10	1.14	2.57	1.10
Vibrionaceae	2.85	1.27	3.07	1.98
Acidaminococcaceae	0.15	0.10	1.34	3.25
Erwiniaceae	2.40	2.01	3.10	3.31
Pectobacteriaceae	3.27	1.47	1.14	0.79
Cellulomonadaceae	2.09	2.43	2.69	2.36
Chroococcidiopsidaceae	0.75	0.75	0.92	0.54
Rhodocyclaceae	0.32	2.80	0.49	0.54
Paenibacillaceae	0.26	0.18	0.42	0.36
Rhodobacteraceae	0.72	0.53	0.89	1.04
Alteromonadaceae	0.79	2.14	1.85	1.22
Pasteurellaceae	0.87	0.66	1.53	1.88
Eubacteriaceae	1.29	0.74	1.49	1.44
Veillonellaceae	1.28	1.61	1.51	1.34
Hafniaceae	1.67	1.28	0.47	0.48
Enterococcaceae	0.42	0.53	0.54	0.36
Streptococcaceae	1.37	0.48	1.22	0.54
Coriobacteriaceae	0.18	1.27	0.10	0.15
Leuconostocaceae	1.09	1.23	0.80	0.85
Porphyromonadaceae	0.88	0.03	0.90	0.21
Tannerellaceae	0.47	1.07	0.29	0.45

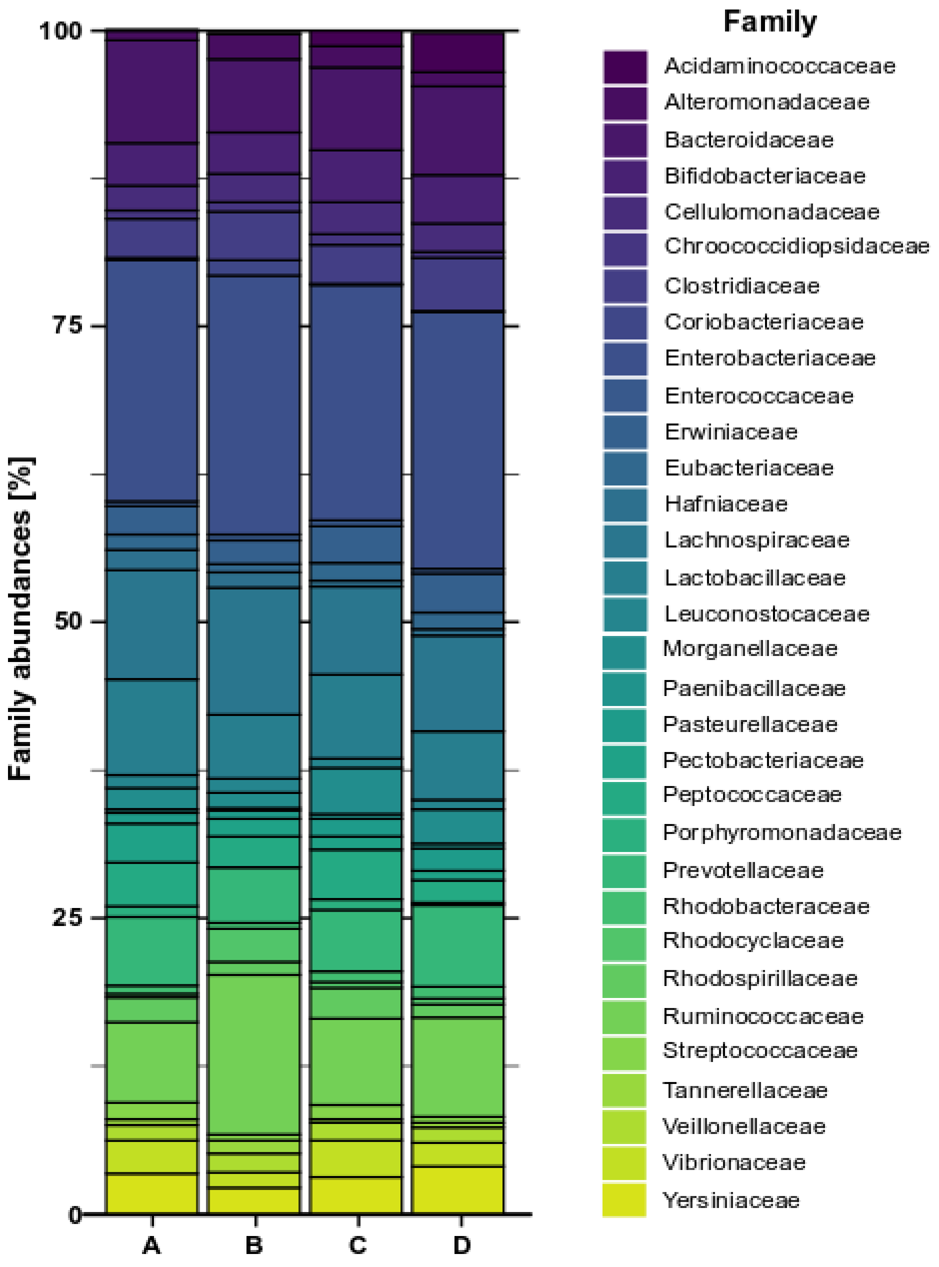

Figure S 9. Relative family abundances based on shotgun metaproteomics.

Table S 16. Bray-Curtis dissimilarity matrix calculated in R using the vegan package. The calculation is based on family abundances observed with shotgun metaproteomics in the colonic communities A, B, C and D.

	**Community A**	**Community B**	**Community C**	**Community D**
**Community A**	1.00	0.77	0.86	0.80
**Community B**	0.77	1.00	0.75	0.75
**Community C**	0.86	0.75	1.00	0.85
**Community D**	0.80	0.75	0.85	1.00

Table S 17. Raw data for riboflavin analysis in the culture supernatant of community A, B, C and D and the culture medium CIM [ng/mL].

CIM	Community A	Community B	Community C	Community D
211	27.7	63.4	31.8	29.0
212	26.8	64.2	30.5	29.6
208	25.0	63.2	32.0	28.7
210	29.3	62.4	29.8	27.3
212	27.8	60.2	29.0	27.3
204	26.1	60.5	28.6	26.2
199	26.3	64.6	28.5	32.0
197	27.0	65.1	27.9	31.1
197	25.7	64.2	28.0	29.5

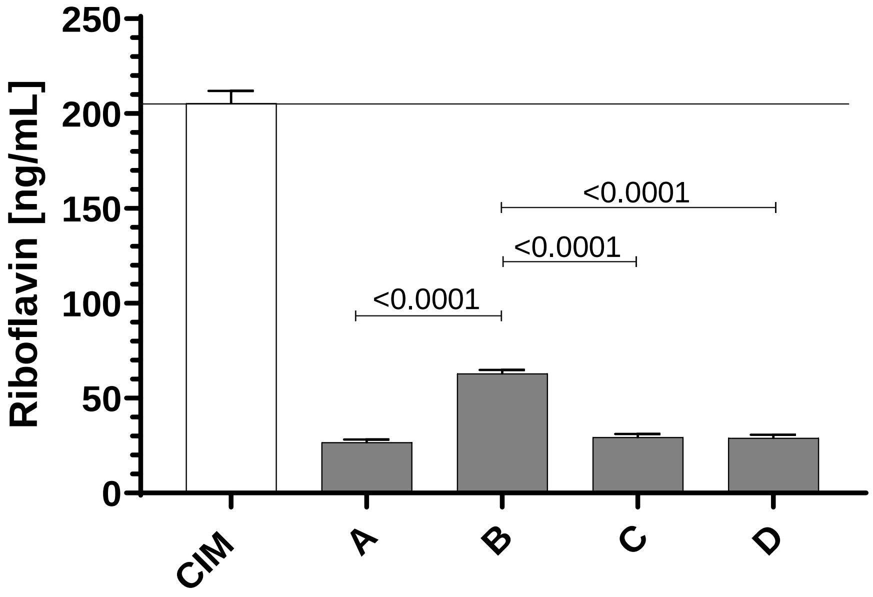

Figure S 10. Riboflavin was quantified in the culture supernatant of the colonic communities A, B, C and D with targeted LC-MS/MS analysis. The communities A, B, C and D showed significantly lower riboflavin concentrations than the culture medium CIM. The riboflavin concentration in community B was significantly higher than in the communities A, C and D (GraphPad Prism, version: 8.3.0, one-way ANOVA, n=3, Tukey correction for multiple comparisons).

Table S 18. Comparison of the riboflavin concentration in the culture supernatant of the colonic communities A, B, C and D and the CIM culture medium. (GraphPad Prism, version: 8.3.0, one-way ANOVA, n=3, Tukey correction for multiple comparisons).

**Sample**	**Significance**	***P***
CIM vs. A	****	<0.0001
CIM vs. B	****	<0.0001
CIM vs. C	****	<0.0001
CIM vs. D	****	<0.0001
A vs. B	****	<0.0001
A vs. C	ns	0.3960
A vs. D	ns	0.6373
B vs. C	****	<0.0001
B vs. D	****	<0.0001
C vs. D	ns	0.9946

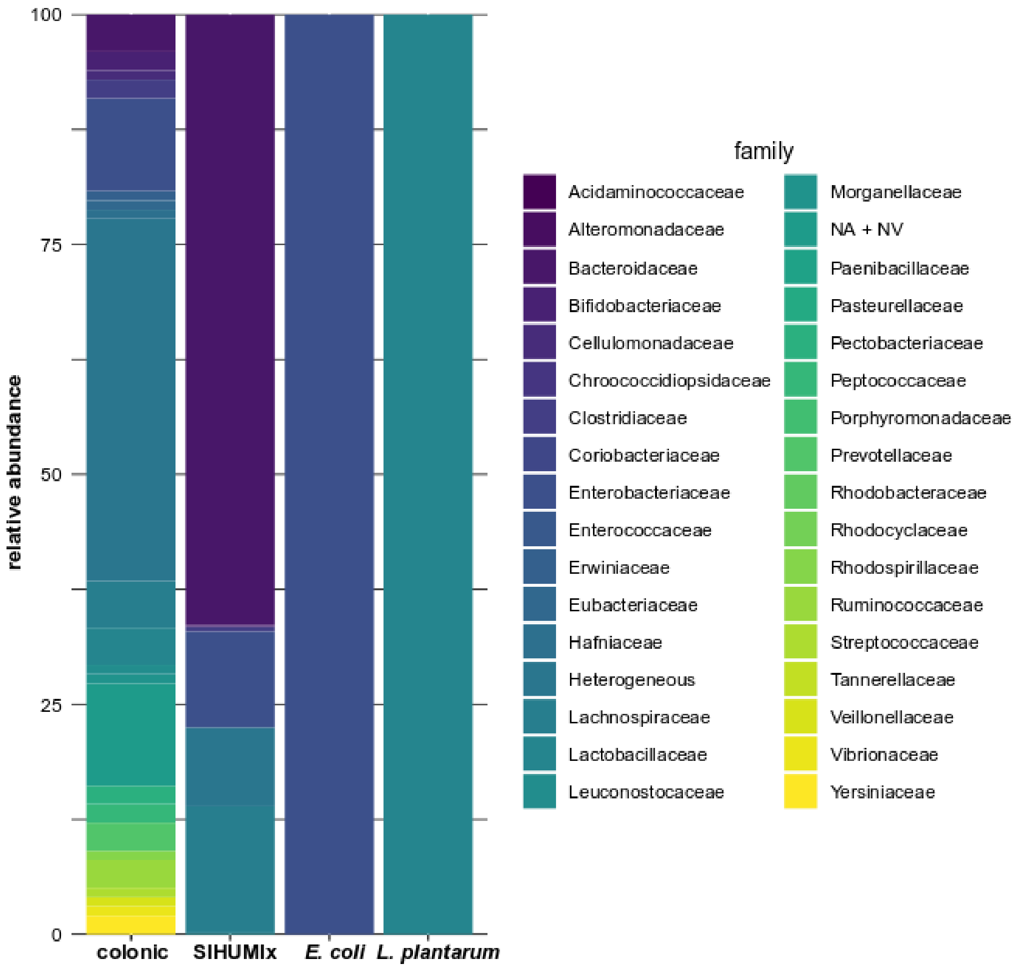

Figure S 11. Analyzing community diversity with 16S rRNA gene analysis. Species richness of a fecal community, a colonic community, the SIHUMIx community and the bacterial strain *E. coli* was compared on the basis of the number of families per sample (Figure 6A).

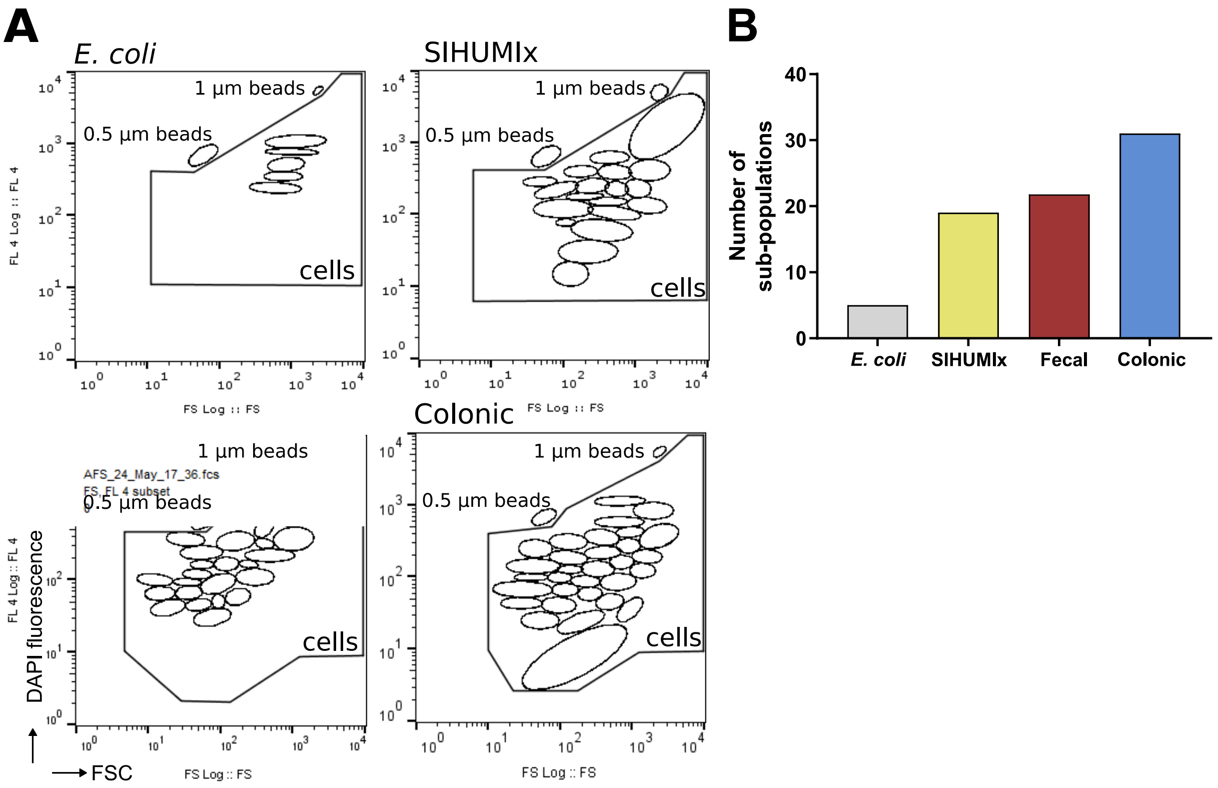

Figure S 12. Master cell gate templates to compare microbial diversity with microbial flow cytometry. (A) Sub-populations were defined in a master gate template for all colonic communities A - D, for all fecal communities and for all SIHUMIx during bioreactor cultivation and *E. coli* cultivated in Hungate tubes. (B) Sample diversity of a fecal human and a swine colonic community, the SIHUMIx community and the bacterial strain *E. coli* were compared based on the number of sub-populations within the cell gate.

Table S 19. Comparison of microbial diversity based on 16S rRNA gene analysis. The number of families identified with 16S rRNA analysis were compared. (GraphPad Prism, version: 8.3.0, one-way ANOVA, n=3, Tukey correction for multiple comparisons).

Sample	Significance	*P*
Fecal vs. Colonic	ns	0.5252
Fecal vs. SIHUMIx	***	0.0008
Fecal vs. E. coli	****	<0.0001
Colonic vs. SIHUMIx	***	0.0002
Colonic vs. E. coli	****	<0.0001
SIHUMIx vs. E. coli	***	0.0002

Table S 20. Comparison of the MAIT cell activating potential of fecal, colonic microbial communities, SIHUMIx and *E. coli*. MAIT cells were stimulated with 200 BpC (n=3). The percentage of activated ($CD69^+/TNF^+$) MAIT cells were compared. (GraphPad Prism, version: 8.3.0, one-way ANOVA, n=3, Tukey correction for multiple comparisons).

Sample	Significance	*P*
Fecal vs. Colonic	ns	0.8732
Fecal vs. SIHUMIx	ns	0.8693
Fecal vs. E. coli	**	0.0018
Colonic vs. SIHUMIx	ns	0.4728
Colonic vs. E. coli	***	0.0008
SIHUMIx vs. E. coli	**	0.0042

Table S 21. Comparing the riboflavin utilization of the fecal and colonic microbial communities, SIHUMIx and *E. coli*. The riboflavin concentration [ng/mL] was measured in the culture supernatant. (GraphPad Prism, version: 8.3.0, one-way ANOVA, n=3, Tukey correction for multiple comparisons).

**Sample**	**Significance**	***P***
Fecal vs. Colonic	ns	0.8721
Fecal vs. SIHUMIx	****	<0.0001
Fecal vs. E. coli	****	<0.0001
Colonic vs. SIHUMIx	****	<0.0001
Colonic vs. E. coli	****	<0.0001
SIHUMIx vs. E. coli	ns	0.6192

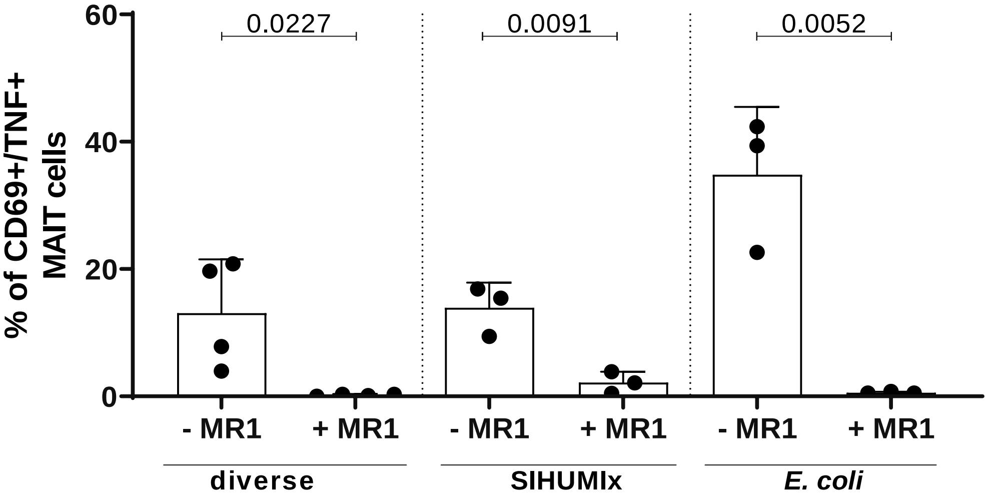

Figure S 13. MAIT cell activation upon stimulation with microbial samples is MR1-dependent. MAIT cells were stimulated with bacteria from diverse microbiota (n=4), with SIHUMIx (n=3) and with *E. coli* (n=3) in the presence (+ MR1) and in the absence of anti-MR1 antibody (- MR1). Bars represent mean ± sd, unpaired t-test.
